# Clinical assessment of a low-cost, hand-held, smartphone-attached intraoral imaging probe for 5-aminolevulinic acid photodynamic therapy monitoring and guidance

**DOI:** 10.1117/1.JBO.28.8.082809

**Published:** 2023-07-21

**Authors:** Shakir Khan, Bofan Song, Srivalleesha Mallidi, Shaobai Li, Hui Liu, M. A. Bilal Hussain, Shaista Siddiqui, Amjad P. Khan, Kafil Akhtar, Shahid Ali Siddiqui, Syed Abrar Hasan, Colin Hopper, Stephen G. Bown, Rongguang Liang, Tayyaba Hasan, Jonathan P. Celli

**Affiliations:** a University of Massachusetts at Boston, Department of Physics, Boston, Massachusetts, United States; b Massachusetts General Hospital, Harvard Medical School, Boston, Massachusetts, United States; c Aligarh Muslim University, Jawaharlal Nehru Medical College, Department of Radiotherapy, Aligarh, India; d The University of Arizona, Wyant College of Optical Sciences, Tucson, Arizona, United States; e Aligarh Muslim University, Jawaharlal Nehru Medical College, Department of Radiodiagnosis, Aligarh, India; f Aligarh Muslim University, Jawaharlal Nehru Medical College, Department of Pathology, Aligarh, India; g Aligarh Muslim University, Jawaharlal Nehru Medical College, Department of Otorhinolaryngology (E.N.T.), Aligarh, India; h University College London, London, England, United Kingdom; i Harvard University and Massachusetts Institute of Technology, Division of Health Sciences and Technology, Cambridge, Massachusetts, United States

**Keywords:** oral cancers, smartphone, intraoral probe, photodynamic therapy, aminolevulinic acid, protoporphyrin IX, fluorescence imaging

## Abstract

**Significance:**

India has one of the highest rates of oral squamous cell carcinoma (OSCC) in the world, with an incidence of 15 per 100,000 and more than 70,000 deaths per year. The problem is exacerbated by a lack of medical infrastructure and routine screening, especially in rural areas. New technologies for oral cancer detection and timely treatment at the point of care are urgently needed.

**Aim:**

Our study aimed to use a hand-held smartphone-coupled intraoral imaging device, previously investigated for autofluorescence (auto-FL) diagnostics adapted here for treatment guidance and monitoring photodynamic therapy (PDT) using 5-aminolevulinic acid (ALA)-induced protoporphyrin IX (PpIX) fluorescence (FL).

**Approach:**

A total of 12 patients with 14 buccal mucosal lesions having moderately/well-differentiated micro-invasive OSCC lesions (<2  cm diameter and <5  mm depth) were systemically (in oral solution) administered three doses of 20  mg/kg ALA (total 60  mg/kg). Lesion site PpIX and auto-FL were imaged using the multichannel FL and polarized white-light oral cancer imaging probe before/after ALA administration and after light delivery (fractionated, total $100\;J/{\mathrm{cm}}^{2}$ of 635 nm red LED light).

**Results:**

The handheld device was conducive for access to lesion site images in the oral cavity. Segmentation of ratiometric images in which PpIX FL is mapped relative to auto-FL enabled improved demarcation of lesion boundaries relative to PpIX alone. A relative FL (R-value) threshold of 1.4 was found to segment lesion site PpIX production among the patients with mild to severe dysplasia malignancy. The segmented lesion size is well correlated with ultrasound findings. Lesions for which R-value was >1.65 at the time of treatment were associated with successful outcomes.

**Conclusion:**

These results indicate the utility of a low-cost, handheld intraoral imaging probe for image-guided PDT and treatment monitoring while also laying the groundwork for an integrated approach, combining cancer screening and treatment with the same hardware.

## 1 Introduction

In this study, we carry out a pilot clinical evaluation of optical technologies for intraoral imaging and photodynamic therapy (PDT). The optical system for image-guided treatment of oral cancers described here is specifically engineered to address challenges faced in resource-limited clinics and is directly motivated by the public health crisis of oral squamous cell carcinoma (OSCC) in South Asia, where widespread chewing of tobacco-based products (e.g., “gutka”) drives truly alarming rates of oral cancer.^1^ In particular, in India, where there is a lack of widespread access to medical infrastructure for cancer management, the rate of new age-adjusted oral cancer cases is 36% (135,929), and the mortality rate is 42.4% (75,290 deaths). The World Health Organization projected that new oral cancer cases would increase to 209,000 (65%) and death would be 116,000 (65%) in India by 2040.^2^ Due to a lack of routine oral examination and timely biopsy, late-stage diagnosis is the norm in India, with 70% of cases presenting at stage III-IV.^3^ As a result, even those patients in rural areas who undergo conventional treatment with surgery and/or chemotherapy show a 5-year survival rate of <50%.^4^ Clearly, there is an urgent need for new technologies that can help enable timely diagnosis and treatment of oral malignancy in challenging, resource-limited rural clinics. Such technologies must be specifically engineered to be produced at low cost, portable, easy to use, and functional in settings with limited infrastructure. In the present report, we demonstrate the integration of previously reported low-cost technology for PDT with an intraoral optical imaging probe previously validated for oral cancer detection and adapted here for image guidance of PDT.^5^^–^^7^

This work builds upon previous development of a low-cost, LED-based intraoral PDT light delivery system, which was found to be safe and effective in treatment of patients with early oral cancer in India.^8^ Briefly, patients were photosensitized by systemic administration of aminolevulinic acid (ALA), leading to accumulation of protoporphyrin IX (PpIX). In addition to serving as a photosensitizer for PDT, PpIX also acts as a fluorescence (FL) contrast agent for tumor imaging and treatment guidance.^9^ We previously reported how a simple smartphone-attached device, consisting of a ring of violet excitation LEDs surrounding an emission filter mounted over the phone camera, could be leveraged as a low-cost solution for imaging PpIX FL in oral lesions.^10^^–^^12^ Although this simple system was leveraged successfully to demarcate lesion positions and estimate the extent of photobleaching after treatment, the physical form factor limited the ability to obtain well-resolved images of lesions inside the oral cavity. Also this use of a single emission further limits the capability to separate PpIX emission from autofluorescence (auto-FL) background. It has been reported previously that analyzing PpIX FL emission relative to tissue auto-FL (red to green channel ratio) improves specificity though the previous single-channel smartphone-based device did not allow for this.^13^^,^^14^

Here we report the adaptation of an oral cancer screening device for use in a new context for intraoral PDT treatment guidance.^15^^–^^18^ This intraoral probe has undergone a pilot phase clinical evaluation e in a small cohort of 12 patients (14 lesions) with early stage oral cancer by obtaining multichannel FL and white light images of the lesion before and after PDT treatment.^15^^,^^16^^,^^19^ This multichannel FL imaging device allows for ratiometric analysis of PpIX FL emission relative to tissue auto-FL, which has been shown to improve specificity.^13^^,^^14^ We examine the quality of FL image segmentation and the capability to enhance lesion margin visualization during PDT compared to standalone auto-FL, white light imaging, and independently obtained ultrasound (US) images. Bringing together complementary technologies for oral cancer screening and PDT in this manner could be significant as part of a broader effort to deliver therapy at the point of care using low-cost technologies, which can be disseminated to rural clinics.^15^^,^^16^^,^^19^

## 2 Materials and Methods

### 2.1 Subject Selection and Imaging Timeline

**Fig. 1:**
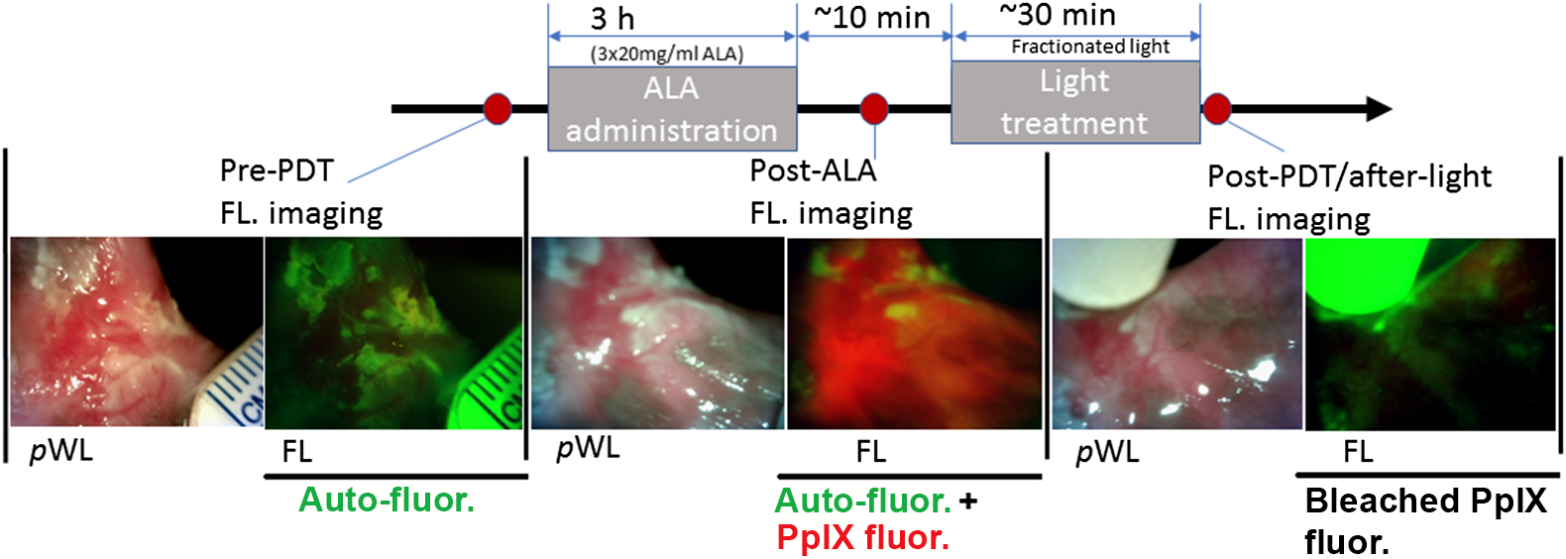
Oral lesion imaging using pWL and FL during ALA-PDT at three timepoints. As shown in this figure, imaging sessions were carried out pre-PDT (immediately prior to ALA administration, for which only background auto-FL is expected), post-ALA (after ALA photosensitization but prior to light delivery, strong PpIX FL signal expected), and post-PDT (after $100\;J/{\mathrm{cm}}^{2}$ light dose, in which point photobleaching is expected.

The intraoral probe was used for imaging buccal mucosal 14 oral lesions (10 patients each with a unilateral lesion, + 2 patients each with a bilateral lesion, 1 female, 11 males, median age: 39 years, age range: 25 to 53 years). The imaging study was conducted in the Departments of Radiotherapy and Clinical Oncology, Jawaharlal Nehru Medical College, Aligarh Muslim University (AMU), Aligarh, India. The PDT trial was approved by the Indian Council of Medical Research and Dana Farber/Harvard Cancer Center Institutional Review Board (Clinicaltrials.gov, identifier: NCT03638622). All oral lesion sizes were <2  cm in diameter with histologically proven $T_{1}M_{0}N_{0}$ OSCC (13 lesions with moderately differentiated OSCC, 1 lesion with well-differentiated OSCC). The study subject had been chewing tobacco products (Gutka, Khaini) for many years (7 to 30 years). Patient recruitment was based on inclusion and exclusion criteria. Patients have no history of photosensitivity toward photoactive compounds, including ALA, and no history of malignant disease treatment. Before the FL imaging, the patient was prescribed antibacterial mouthwash (chlorhexidine gluconate 0.2% w/v) for 1 week to decrease the bacterial load at the lesion site and reduce the microbial FL.^20^^,^^21^ White light and auto-FL images were taken before the first oral dose of ALA (DUSA Pharmaceuticals, Inc., Wilmington, Massachusetts, United States), 20  mg/kg dissolved in fruit juice. The second and third doses of ALA (20  mg/kg each) were given at hourly intervals. Second PpIX FL imaging was taken after the third dose of ALA (Fig. 1). The fractionated schedule of oral ALA administration using three smaller dosesat hourly intervals was chosen to mitigate side effects based on previous literature.^22^ In a larger patient cohort receiving the same dosage reported previously, one patient experienced hypotension, though it was resolved through hydration.^22^^–^^24^ No post-PDT skin sensitivities were reported though we did observe minor abnormalities in liver and renal function tests after administering ALA, but these quickly resolved without any intervention. Light from an LED source (635 nm peak) was delivered to the lesion surface (total dose $100\;J/{\mathrm{cm}}^{2}$ at maximum irradiance $48.7\;\mathrm{mW}/{\mathrm{cm}}^{2}$) using modular 3D-printed fiber-coupled applicators to facilitate accurate and ergonomic illumination of the target tissue.^6^^,^^8^ The total duration of light delivery was 34 min with two fractions and 2 min intervals in between. The beam spot intensity was uniform to within about 10% variation from center to edge and previous cell culture validation experiments showed that with these dose parameters beam spot size correlates almost perfectly with zone of cell killing.^5^ The third PpIX FL image was taken after PDT treatment to measure PpIX and auto-FL bleaching (see data S2 in the Supplementary Material).

### 2.2 Intraoral Device

**Fig. 2:**
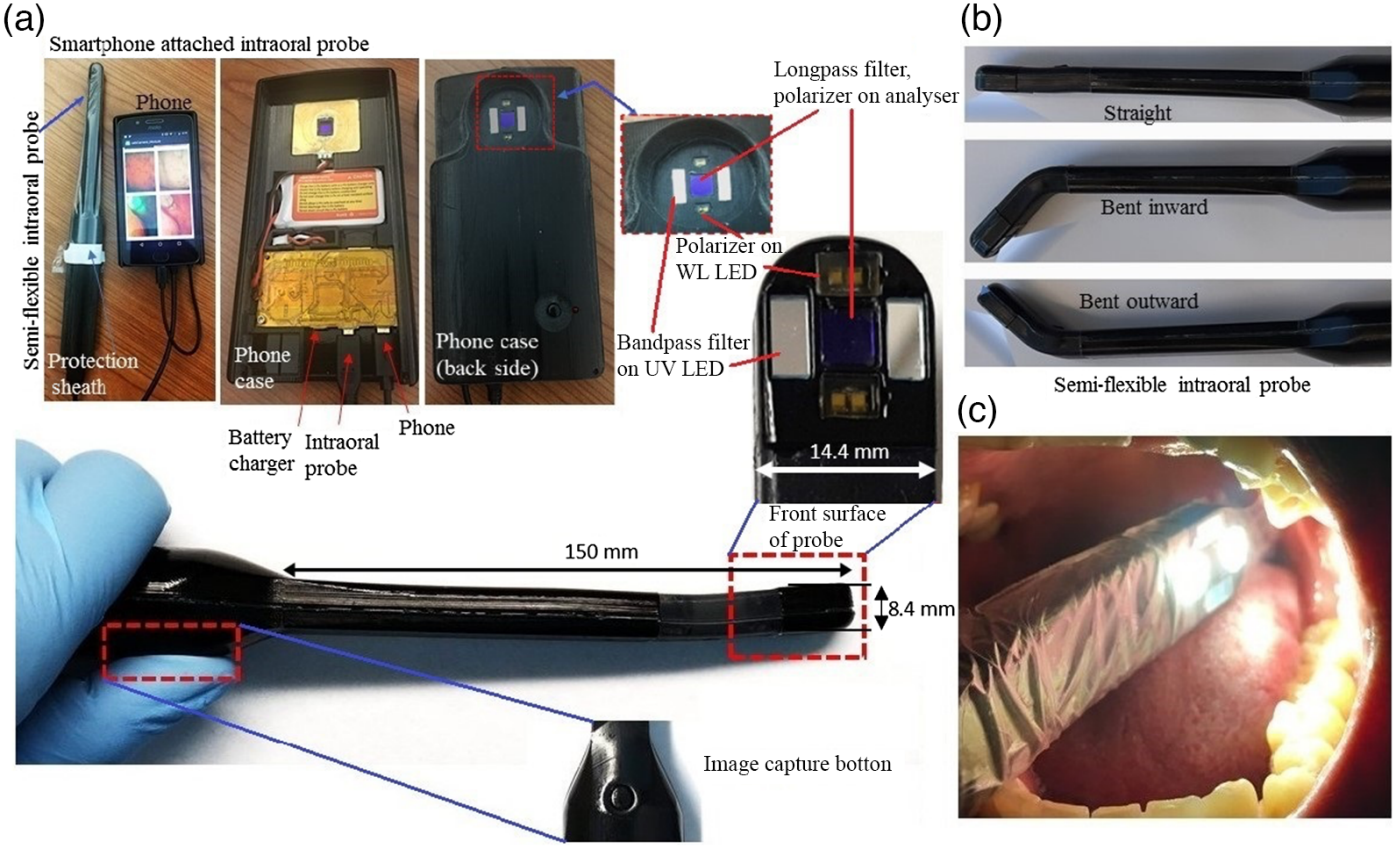
The intraoral imaging probe and attached smartphone. (a) The probe has a semi-flexible intraoral probe head with a camera connected to an MotoG android smartphone via USB. Additionally, another identical camera is installed on the backside of the phone case for whole-mouth imaging. (a), (c) To maintain hygiene during patient testing, the probe is covered with a hygienic sleeve (TIDI Products, Neenah, Wisconsin). (b) The flexible intraoral probe can be adjusted to bend up to 45 deg in either direction, allowing for lesion location examination in anterior, posterior buccal mucosa, and near/at retro-molar trigone.

The intraoral probe used here was originally developed for oral cancer screening, and it was successfully tested for this application on 5000 patients.^15^^–^^18^ In previous studies, the device used only white light and auto-FL imaging modality for the screening using 405 nm light excitation on the lesion. The auto-FL contrast is enhanced by short-pass filters (425 nm, Asahi Spectra, Tokyo, Japan) and a long-pass filter (470 nm, Asahi Spectra) in front of the UV LED and camera since auto-FL wavelength of regular buccal mucosal tissue is ∼500  nm, whereas premalignant and malignant buccal mucosal tissue exhibit auto-FL wavelengths ranging from 635 to 705 nm.^25^^,^^26^ Orthogonal linear polarizers (Edmund Optics, Barrington, New Jersey, United States) are placed in front of the camera module and white LEDs, which results in a polarized white light (pWL) image. This device is the third generation of hardware having a dual-mode, wide field of view for FL imaging and pWL imaging. Device components include a commercial smartphone (Moto G5 Android), a handheld intraoral imaging probe, a light-emitting diode driver, a smartphone case attached to a rechargeable lithium battery, and mobile application software (Fig. 2). Both the back of the smartphone case and attached intraoral probe have four 405 nm Luxeon UV U1 LEDs (Lumileds, Amsterdam, Netherlands) for FL imaging and four 4000-K Luxeon Z ES LEDs for pWL imaging. PpIX has broad Soret band absorption around 410 nm and is simultaneously excited along with lesion site auto-FL. The uniformity of illumination was measured to be 83.80% for pWL and 89.84% for UV light. The probe head has a horizontal aluminum stiffener/heatsink that sits between its two halves, which is securely clamped in place. This helps to keep the probe stable and connected, along with the screw capture notches. To minimize the duration of white light exposure and photobleaching of PpIX and intrinsic auto-FL, both pWL and UV light have a minimum exposure time of 30 ms. To prevent the transmission of oral infections, a sterile sleeve (TIDI Products, Neenah, Wisconsin, United States) was utilized during the imaging of oral lesions. This sleeve was placed over the intraoral probe surface to prevent any contact with the buccal mucosal surface, as demonstrated in Fig. 2. During imaging, the distance between the intraoral probe and the lesion surface was nearly constant (20 to 25 mm), and intensity signals were the same within 20 to 25 mm range. This constant distance is attributed to the probe’s working distance of 20 mm and a narrow depth of field of 5.0 mm, where the smartphone-attached probe gets the sharpest imaging at ∼20  mm from the lesion surface.

The long-pass filter in front of the CMOS sensor, blocks the excitation light around 405 nm, enabling it to get the lesion-generated PpIX FL in our PDT study. The existing mobile imaging system has two imaging mode options with camera (Omnivision OV5648 integrated CMOS image sensor): (1) intraoral imaging with the flexible intraoral probe (with a front view of the phone case) and (2) whole mouth imaging with the phone camera (back view of the phone case). This semi-flexible intraoral probe has increased clinical ergonomics. It can be bent to access all areas of the oral cavity where the probe is straight, bent for inward imaging and outward imaging (Fig. 2). The silicone (Mold Star 20Tand Black Silc Pig, Smooth-On, Macungie, Pennsylvania, United States) section of the probe provides flexibility up to 470% elongation before breaking. A 250  μm thick, 11 mm wide piece of aluminum sheet metal is embedded in the flexible section to maintain the probe head angle after bending to the desired position. It also serves as a thermal sink for the illumination LEDs. Each generated RGB image has the property of 1344×1792  pixel dimension and 8-bit depth. Due to its simplicity in imaging, this device could be easily used by a non-specialist in rural areas equipped with low-resource clinical settings.

### 2.3 In Vitro, Intraoral Device-Based Protoporphyrin IX Fluorescence Imaging

The device was calibrated on the head, and neck cancer OSCC cell line TR146 (cat. no. ECACC 10032305, source histology: well-differentiated keratinizing squamous cell carcinoma), which were used for initial *in vitro* imaging studies. Here TR146 cells were cultured in two $75\;{\mathrm{cm}}^{2}$ T-flasks at 37°C in Ham’s F-12K (Kaighn’s) medium containing L-glutamine and sodium bicarbonate buffer system and supplemented with a 10% FBS, 100  μg/ml penicillin/streptomycin, 0.5  μg/ml amphotericin-B. The cells were trypsinized (trypsin 25%) and harvested when cell density was achieved around $0.5\times 10^{6}\text{ cells}/\mathrm{ml}$ in each T-flask. Cells were incubated with 3 mM ALA (5-ALA HCI, Sigma Aldrich, Israel) for 4 h in a new T-flask. ALA incubated cells were pellet down after 5 min centrifugation at 5000 rpm. TR146 generated PpIX FL images were taken in disposable polymethyl methacrylate (PMMA or “acrylic”) cuvettes, where aggregated cells (dissolved in ${\mathrm{TiO}}_{2}$) were pipetted down in 2% (w/v) sodium alginate (SA) hydrogel phantom (crosslinked by DPBS supplemented with ${\mathrm{Ca}}^{+2}$ and ${\mathrm{Mg}}^{+2}$ ions). PpIX FL intensities were calibrated at various concentrations of PpIX (0 to 50  μg/ml) in ${\mathrm{TiO}}_{2}$ (0.58  g/l) tissue phantom.^7^^,^^27^^,^^28^

### 2.4 Lesion Image Segmentation

Images were saved as an 8-bit unsigned integer RGB image and split into red and green channels (using Python NumPy module), where dominantly green channel represents the auto-FL, and red channel represents PpIX FL.^13^^,^^14^ A lesion with margins and non-malignant surrounding tissue was segmented on relative red (PpIX) and green (auto-) FL intensity (R-value, range: 0 to 4). The factor of 4 was arbitrary to expand the R-value scale over a larger range. The red ($I_{\text{redNorm}}$) and green ($I_{\text{greenNorm.}}$) pixel intensity were normalized on total RGB intensity at the position of (x,y)th pixel ($I_{\text{total}}$ (x,y)) [Eqs. (1)–(3)]. The Python OpenCV package was used for FL and R-value image segmentation^29^ (see image processing Python codes and methods in the Supplementary Material):

$$I_{\text{total}@x,y}=I_{\text{red}@x,y(\text{pix value range}:\;1\text{ to }255)}+I_{\text{green}@x,y(1\text{ to }255)}+I_{\text{blue}@x,y(1\text{ to }255)},$$ (1)

$$I_{\text{greenNorm.}}=\frac{I_{\text{green}@x,y}(1\text{ to }255)}{I_{\text{total}@x,y}},$$ (2a)

$$I_{\text{redNorm.}}=\frac{I_{\text{red}@x,y}(1\text{ to }255)}{I_{\text{total}@x,y}},$$ (2b)

$$R_{\text{value}@x,y(\text{range}:\;0\text{ to }4)}=4*\frac{I_{\text{redNorm.}}}{I_{\text{greenNorm.}}}.$$ (3)

### 2.5 Statistical Analysis

The central value of each lesion site and *in vitro* parameters (i.e., red, green FL parameters, correlation of ${\mathrm{TiO}}_{2}$ phantom produced PpIX FL intensity-concentration) were analyzed using R (Comprehensive R Archive Network).^30^ The difference between mean values of red and green intensities and their relative values during three-time points, pre-ALA, post-ALA, and post-light, was assessed using the Kruskal Wallis (KW) test, an alternate one-way ANOVA test. The Cleveland dot plot was drawn to reduce the clutters of the bar graph using the ggplot2-based “ggpubr” package in the R console. The significance values are considered by calculating the P-values at: ***p<0.001, **p<0.01, and *p<0.05.

## 3 Results and Discussion

### 3.1 Device Performance Using Tissue Phantoms

**Fig. 3:**
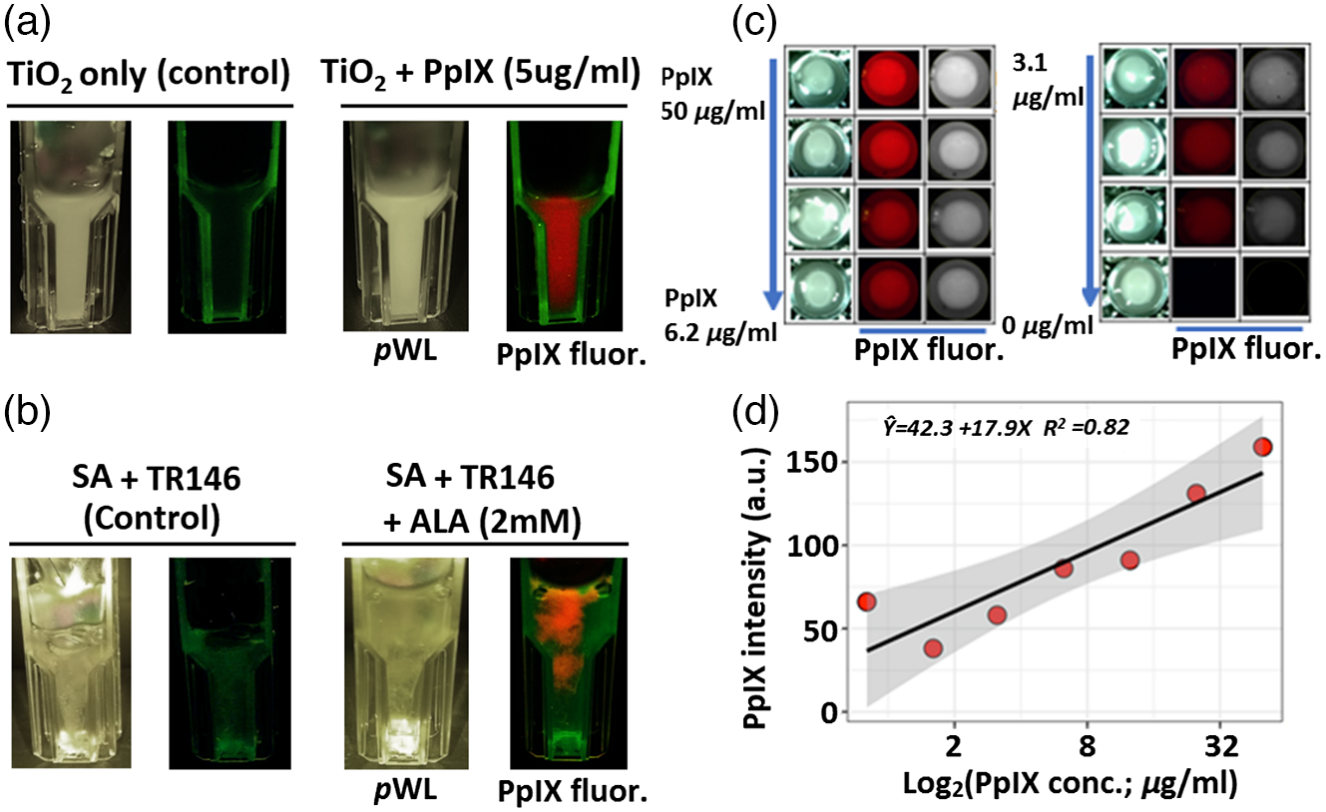
PpIX FL *in vitro* using imaging ${\mathrm{TiO}}_{2}$ tissue phantom and 2% SA hydrogel. (a) Imaging of PpIX in tissue phantom ${\mathrm{TiO}}_{2}$. (b) Imaging of OSCC TR146 produced PpIX in SA hydrogel. (c), (d) Calibration of PpIX FL intensity at various concentrations of PpIX in ${\mathrm{TiO}}_{2}$.

We initially assessed the intraoral device in the laboratory using PpIX phantoms (${\mathrm{TiO}}_{2}$ and SA) and a cell culture-based tissue model (ALA photosensitized TR146 cultures). The TR146 cell line was used as a tissue phantom model since it has been well characterized and previously applied for constructing an *in vitro* human buccal epithelium model for buccal drug targeting.^31^ Here we also used ${\mathrm{TiO}}_{2}$ as a well-established tissue phantom.^27^ Liquid ${\mathrm{TiO}}_{2}$ is a scattering agent and recapitulates a similar wavelength-dependent scattering coefficient as in oral malignant tumor tissue ($\mu_{s}^{\lambda =630}$: $9.4\;{\mathrm{cm}}^{-1}$).^32^ We took images just after stirring the ${\mathrm{TiO}}_{2}$ solution as it has the suspension form in most of the media. The range of concentration PpIX (0 to 50  μg/ml) in ${\mathrm{TiO}}_{2}$ (0.58  g/l) was based on physiologically generated PpIX after ALA administration in the human body.^28^ At a lower concentration of PpIX (5  μg/ml), the intraoral probe detects the red FL in the cuvette [Figs. 3(a) and 3(b)]. TR146 aggregated and dissolved in SA phantom showed the generated PpIX FL. The cuvette wall on edge departs the green auto-FL and is differentiated with contrast in FL signal from non-ALA treated controls having only untreated TR146 in alginate hydrogel medium. The PpIX FL intensity signal increases linearly with increasing PpIX concentration. The regression correlation shows the robust coefficient of determinant ($R^{2}$) value within a 95% confidence interval (gray confidence band on the plot) [Figs. 3(c) and 3(d)].^33^^,^^34^

### 3.2 Clinical Evaluation of Intraoral Probe by PpIX, Autofluorescence, and Ratiometric Images

**Fig. 4:**
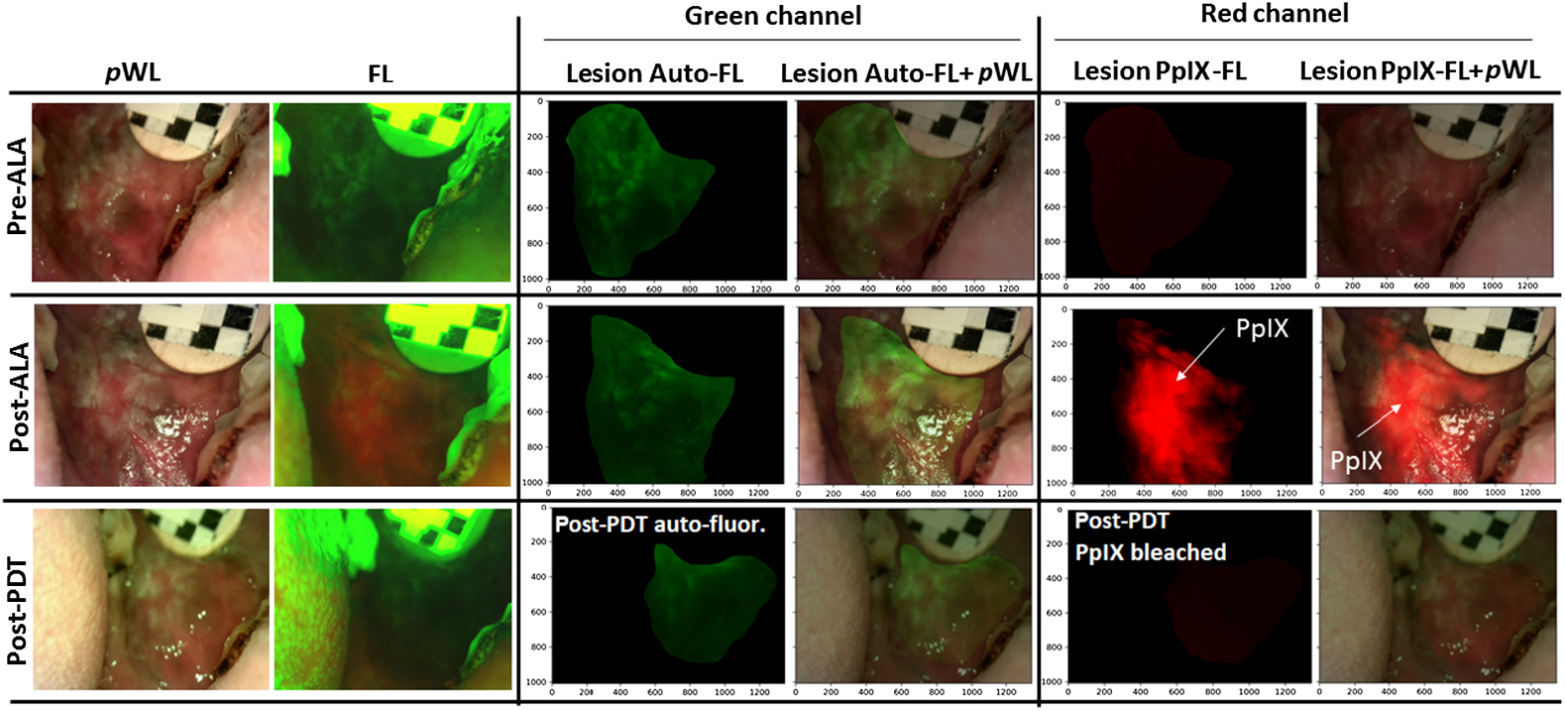
Image segmentation of FL and white light images (pre-ALA, post-ALA, and post-PDT). The red and green channels correspond largely to PpIX FL and auto-FL, respectively.

In general, it is difficult to differentiate cancerous from healthy buccal mucosa by traditional clinical oral evaluation (COE) with white light. The reported sensitivity of COE is 93%, though specificity is only 31% due to a high number of false positives.^35^ To improve specificity, we combined lesion-specific PpIX (90% sensitivity and 60% specificity)^36^ and auto-FL (variation in reported 30% to 100% sensitivity, 6% to 100% specificity)^37^ to increase specificity and sensitivity as compared to either alone signal.^13^^,^^14^^,^^38^^,^^39^ This feature, combined with the tendency of PpIX to accumulate in malignant tissue leads to an elevated ratio of red to green FL emission in malignant versus normal tissues. As such, ratiometric measurement of both PpIX and auto-FL has been shown to improve the accuracy of distinguishing malignant lesions.^13^^,^^14^ Moreover, the ratio has been shown to correlate with the progression of premalignant lesions from mild to severe dysplasia.^13^^,^^14^^,^^40^^,^^41^ With our RGB multichannel device the green channel is predominantly auto-FL, the red channel represents PpIX FL (Fig. 4). The auto-FL attributed to normal tissue’s intrinsic fluorophores, such as collagen, flavin adenine dinucleotide (FAD), nicotinamide adenine dinucleotide (NADH), and elastin. The combination of these fluorophores produces a broad FL spectrum after the near UV excitation (300 to 450 nm).^42^ Several clinical and non-clinical studies demonstrated the decreased auto-FL intensity in the tumor tissues compared to normal tissues. In particular, collagen and elastin contain aromatic amino acids that give them strong FL linked to how cells and tissue are structured as connective tissue under the epithelium. The progression of mucosa to premalignant and malignant stages involves a gradual breakdown of the collagen crosslinking associated with the carcinogenesis. This breakdown leads to a decrease in collagen FL. Additionally, mitochondrial fluorophores (NADH and FAD) and endogenous porphyrin are related to metabolic processes in mucosal epithelial tissue.^43^^–^^45^ Studies have shown that as epithelial cells progress from premalignant to malignant, their tissue metabolic activity increases. This leads to a rise in NADH FL and a decrease in the redox ratio (FAD/NADH + FAD).^46^^–^^48^

In this study, we utilized an excitation wavelength of 405 nm to detect a combination of NADH and FAD signals, which produced an FL peak ranging from 490 to 520 nm.^49^ However, according to the study, 405 nm excitation did not produce the collagen signal to predict the tissue structure, as stromal collagen predominantly absorbs light at around ∼355  nm.^49^ Using the 405 nm excitation, we avoided confusing keratin FL signals, which can often resemble collagen signals.^49^^,^^50^ This is important because keratinization-related lesions can produce false collagen signals in premalignant and malignant tissues.^51^^,^^52^ This auto-FL property has been exploited in demarcating a lesion with malignant, non-malignant, oral dysplasia (mild to severe dysplasia).^18^^,^^53^^,^^54^ It is important to note that the epithelial auto-FL (of FAD, NADH) and PpIX FL observed are measured only on the tissue surface as 405 nm excitation light used here reaches a depth of only about 1 mm in mucosal tissue.^55^

After ALA administration, the red channel shows strong PpIX FL (Fig. 4). The bleaching of PpIX FL after PDT visually confirms the area of light delivery, including surrounding margins. In contrast, auto-FL decreases after ALA administration, but intrinsic fluorophores also bleached after light delivery (see data S1 in the Supplementary Material). In interpreting the photobleaching data, it is important to again recognize that the blue/violet excitation used here has limited penetration and as such is sampling a small volume relative to the treated volume using red light with penetration of several millimeters. For example, Dimofte et al.^56^ noted that light delivery for ALA PDT treatment of similar head and neck lesions using a nearly identical excitation wavelength around 635 nm achieves penetration of about 3.4 mm. As a result, the assessment of photobleaching is highly biased toward the tissue surface in the measurements reported. However, as noted above, the use of 405 nm light in this study also played a critical role in excitation of auto-FL, which is used to enhance selectivity of tumor imaging. It is also worth noting that the hardware used here does not allow for measurement of tissue optical properties, which have been shown by others to change during PDT in similar lesions.^57^^,^^58^

**Fig. 5:**
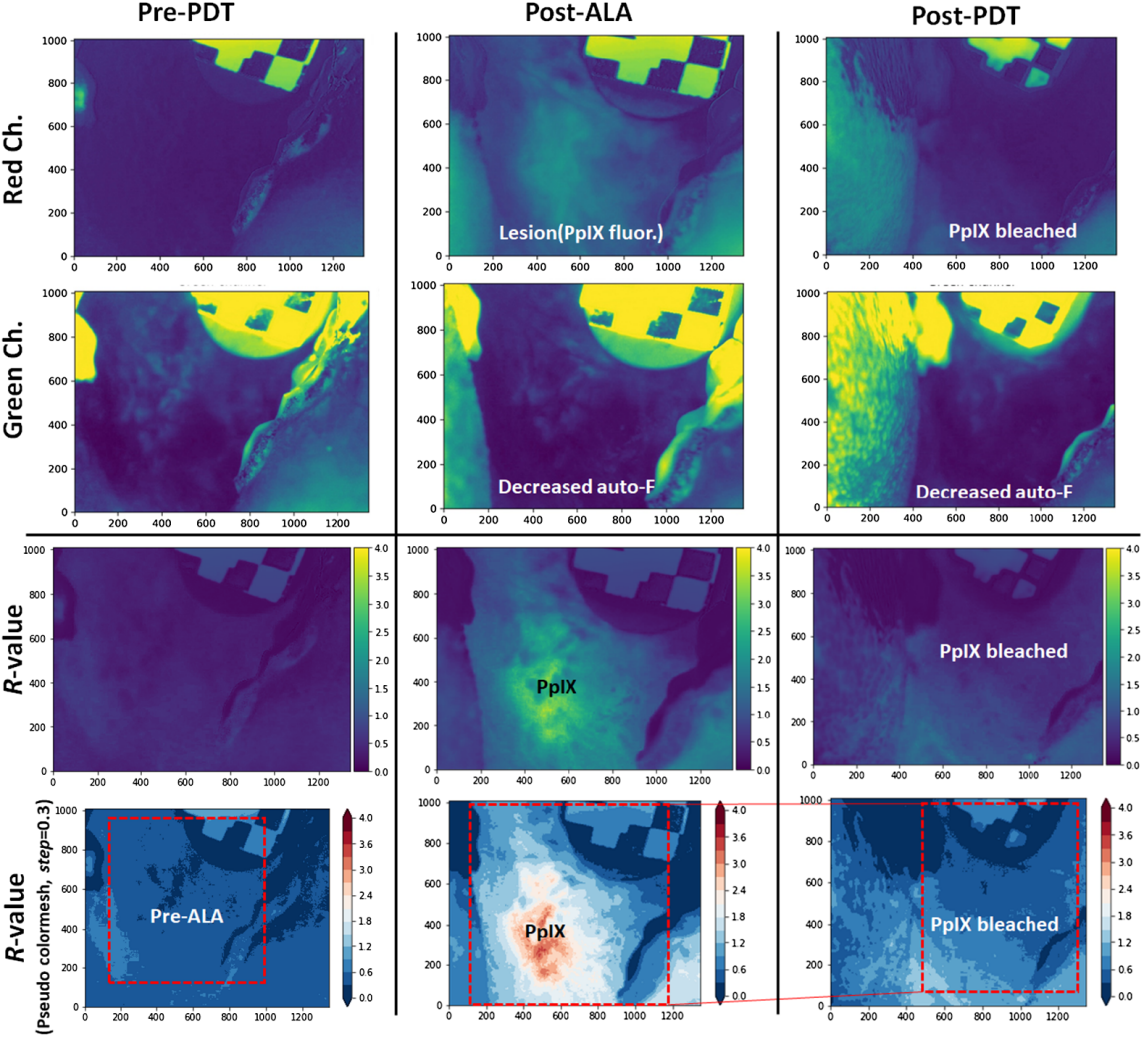
Ratiometric analysis of multichannel FL images acquired before photosensitization (Pre-ALA), after photosensitization (post-ALA), and after therapeutic light delivery (post-PDT). Red channels show the lesion site PpIX FL and post-PDT PpIX bleaching. The auto-FL signal in the green channel significantly decreases in both post-ALA as well as in post-PDT as compared to pre-PDT. Ratiometric R-value (in the range of 0 to 4) explicitly shows the PpIX FL with clear margins. Further, pseudo-colormesh R-values with steps = 0.3 was used for image segmentation and lesion site R-value quantification.

**Fig. 6:**
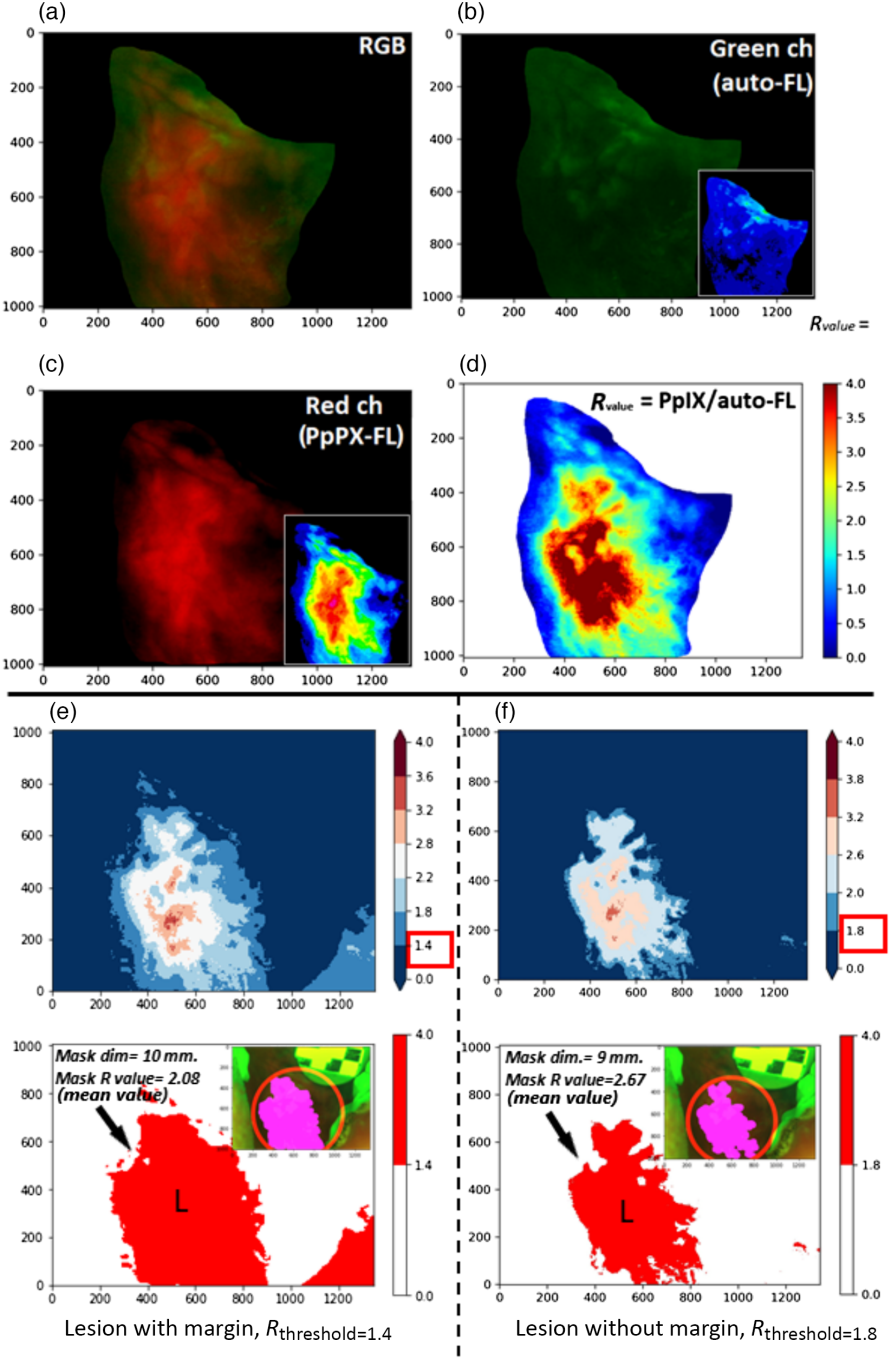
Segmentation of images acquired using the intraoral probe after ALA photosensitization. FL image (a) is split into red (PpIX FL) and green (auto-FL) channel images with pseudo-color 16LUT, (b), (c) inset. (d) The relative R-value visualized in the Python color map (cmap) displays the pseudo “jet” colors corresponding to R-values. (e), (f) The R-value thresholding at 1.4 and higher-end 1.8 show the masking of lesions with 10 to 9 mm diameter size. The red-colored masked regions show corresponding mean R-values.

Further, based on relative FL pixel intensity (denoted as R-value), the image segmentation shows excellent demarcation of lesion FL (Figs. 5 and 6). The increased R-value corresponds to a relative increase in PpIX FL and a reduction of auto-FL intensity [$I_{\text{redNorm}}/I_{\text{greenNorm}}$, Eq. (3)]. In the earlier studies, the relative FL intensity of split channels (R, G, B) predicts the malignant and normal tissue.^40^^,^^41^ Sharwani et al.^14^ differentiated not only malignant tissue but also oral premalignant lesion stages, such as dysplasia, hyperplastic tissue, and inflammation, where dysplastic tissue and carcinoma *in situ* (CIS) showed a higher R-value with 83% to 90% sensitivity and 79% to 89% specificity. The Sharwani et al.’s study set the threshold value of R-value at 1.2 to differentiate the normal to dysplastic/CIS tissue, but in our image segmentation, threshold value bar (R-value) was set at 1.4, which also includes the normal margin (non-malignant/dysplastic) in addition to the lesion (Fig. 6). This low-threshold value segmentation guides the PDT light applicator on the lesion surface with a margin for targeted light dosimetry (see data S2 in the Supplementary Material: 14 lesion’s R-value images and R-value range: 1.65 to 3.11).

**Fig. 7:**
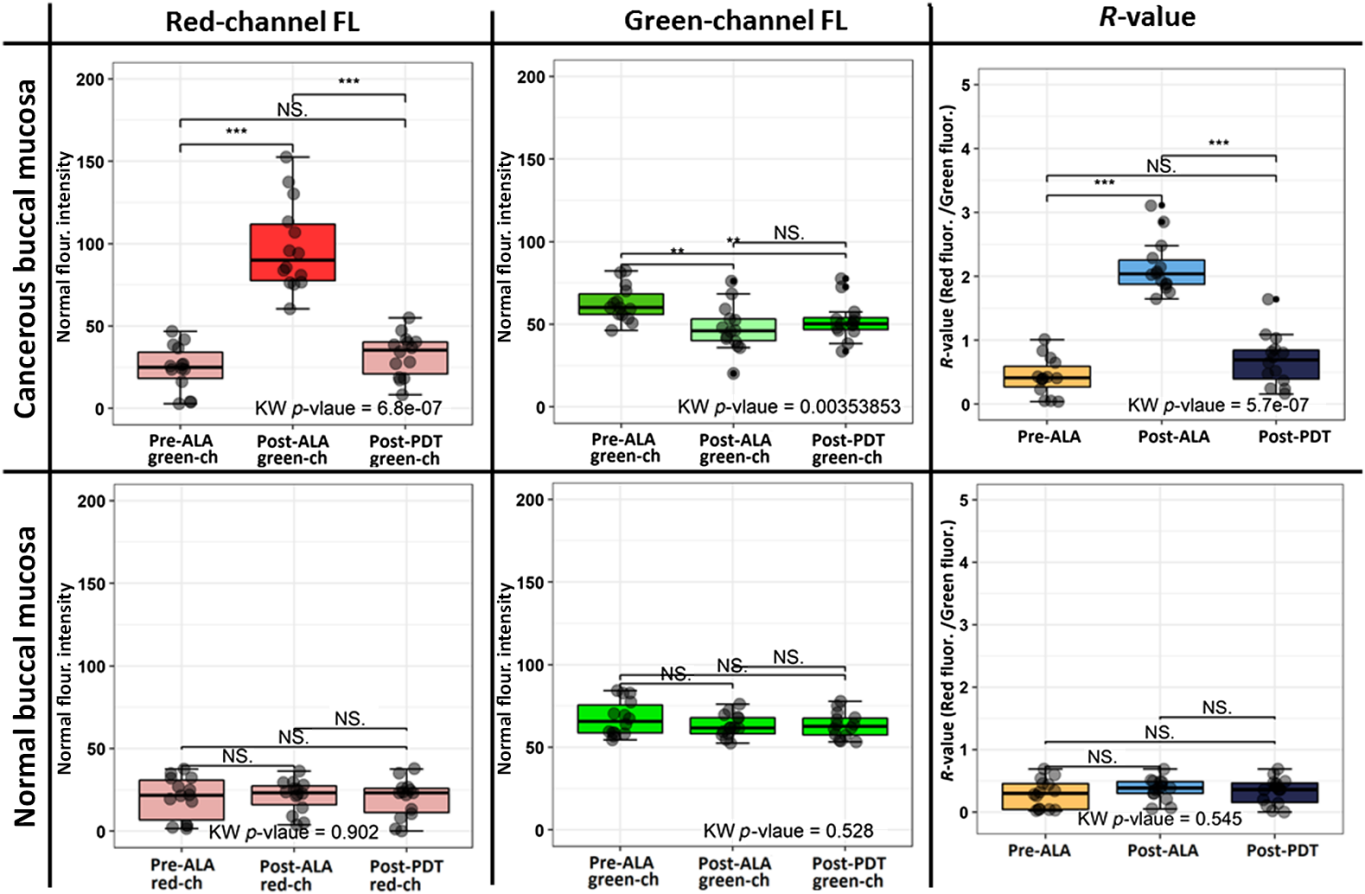
Box plot of normal FL intensities pre-PDT, post-ALA, post-PDT (red, $I_{\text{redNorm}}$; green, $I_{\text{greenNorm}}$; and relative FL intensities, R-values) of cancerous lesions (n=14) and non-cancerous healthy buccal mucosa tissue. Significant differences were determined by the KW test (p-value: ***p<0.001, **p<0.01, and *p<0.05).

The post-ALA images showed a significant decrease in auto-FL. This intrinsic FL (auto-FL) tried to recover as pre-ALA with little gain where the R-value difference (ΔR-value=R-value [Pre-ALA − Post-PDT]) is non-significant (Fig. 7). The post-ALA R-value of the cancerous lesions was 2.23 (mean) as compared to normal tissues (mean = 0.68), which indicates the cancer lesion specific PpIX synthesis and less auto-FL. This difference between R-value among pre-ALA and post-ALA is very significant (p<0.001) as compared to the normal buccal mucosa, where R-value at three-time points (pre-PDT, post-ALA, and post-PDT) are almost identical with no significant difference (range = 0.29 to 0.33). The photobleaching manifested by a reduction of the R-value from post-ALA to post-PDT by 3.2 times. The change in red-channel FL intensity confirmed the total bleaching of PpIX by 3.1 times ($I_{\text{redNormpost-}\mathrm{ALA}}/I_{\text{redNormpost-}\mathrm{PDT}}$).

### 3.3 Comparison of Fluorescence with Ultrasound

**Fig. 8:**
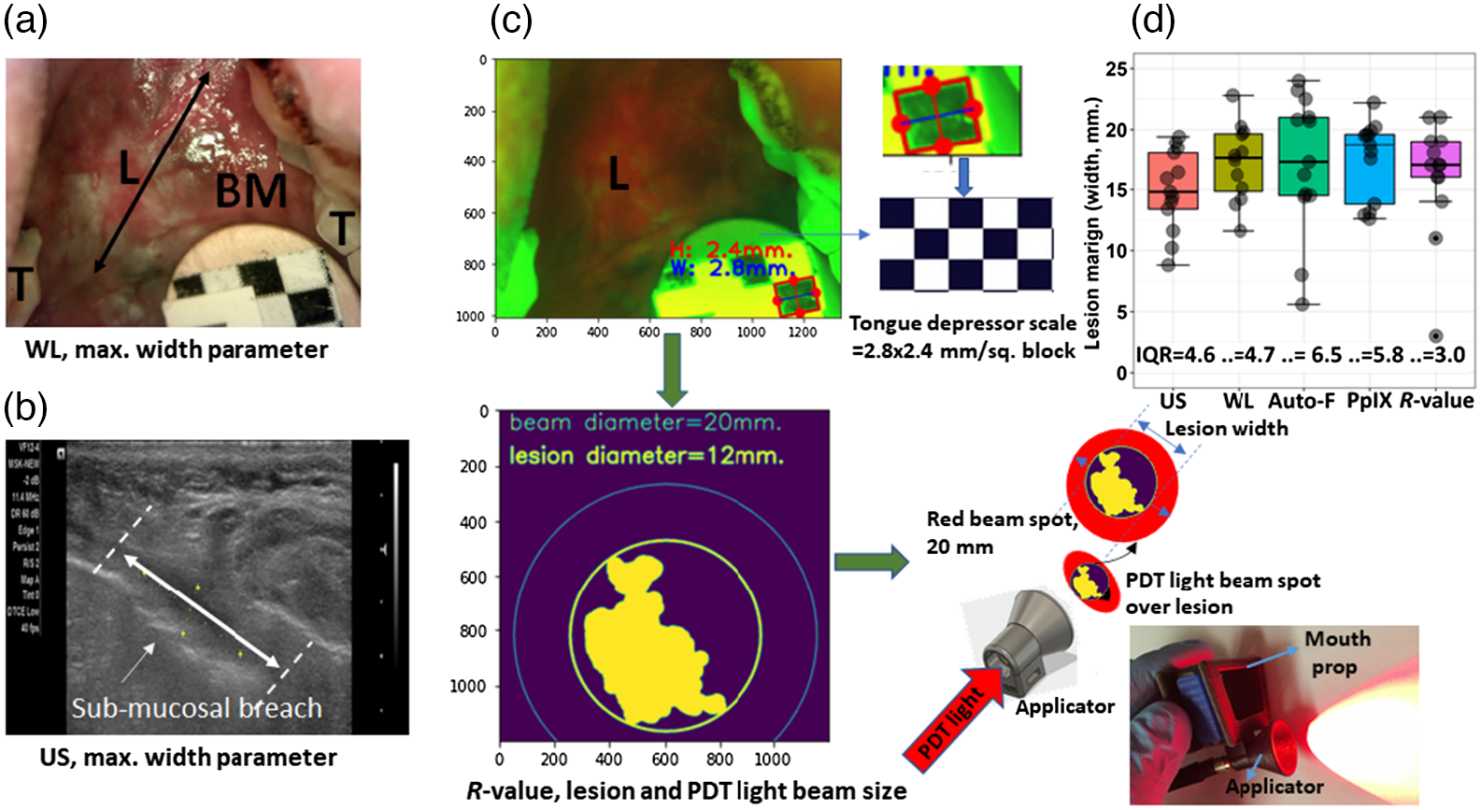
(a)–(c) Comparison of lesion size measured by pWL, PpIX FL, and US. The checkered reference scale predicts the lesion diameter, which is covered by PDT light source beam spot (20 mm.) on lesion. Here PDT light delivered by 3D-printed intraoral applicator. (d) Box plot of distribution of lesion width among 13 lesions with IQR.

**Fig. 9:**
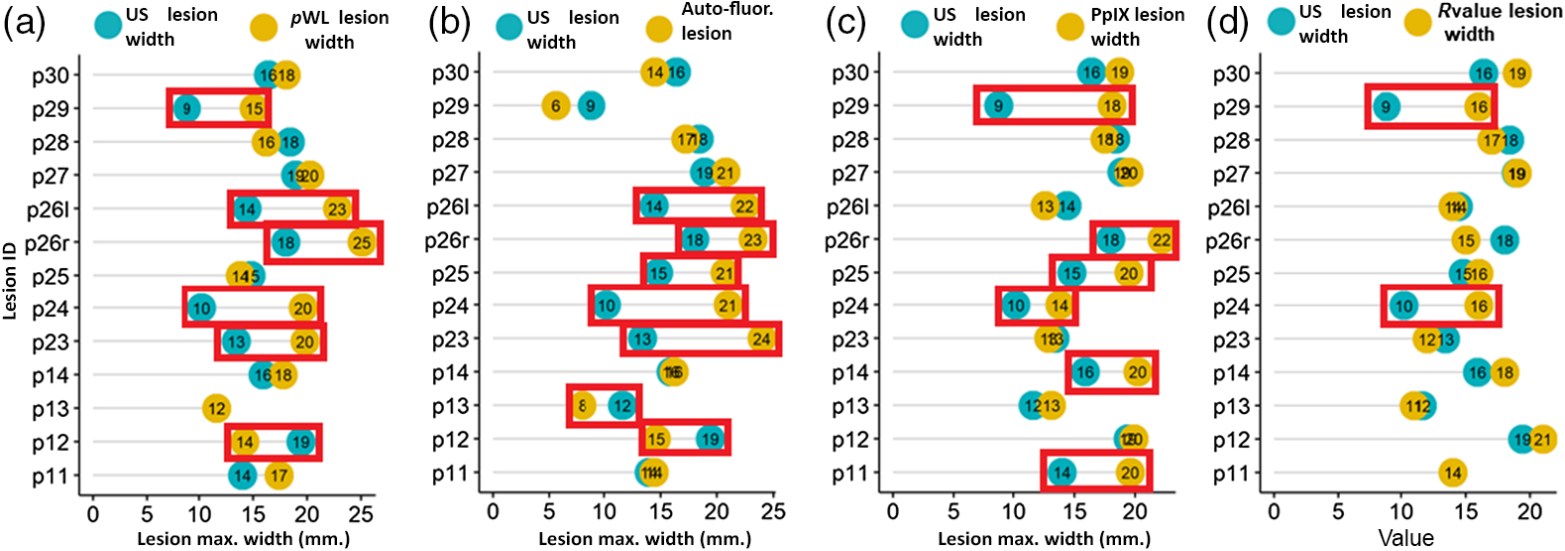
(a)–(d) Cleveland dot plot comparisons of the maximum width of lesion measured by pWL, PpIX FL, and US. The dot plots show lesion width among four pairs of pWL, US, auto-FL, PpIX, and segmented R-value. The marked red box represents the significant difference in lesion width parameter measurement (i.e., >3  mm.).

In order for PDT to achieve a complete tumor response, it is essential that light delivery fully covers the lesion surface with healthy margins. In our clinical study, PDT light delivery used a 3D-printed intraoral applicator-prop applied on the lesion surface. The red LED light from the applicator produces a 20-mm red spot encompassing the lesion (lesion size; <20  mm, $T_{1}M_{0}N_{0}$ stage) (Fig. 8). PDT light covers the lesion with healthy margins and delivers the calculated light dose. The lesion size parameters were also established by prior US imaging, where the maximum width of 14 lesions is <20  mm. Here we compare the FL lesion image-based margin parameter with US as it is reported as a gold standard for primary tumor detection with sensitivity and specificity ranging from ∼75% to 100%.^59^ The maximum lesion width parameter is quantified using pWL, auto-FL, PpIX FL, and R-value segmentation. The boxplot of auto-FL showed a wide range distribution of lesion width [interquartile range (IQR), 6.5] [Fig. 8(d)] in contrast to the R-value segmentation distribution (IQR, 3.0). However, the difference in the mean values was insignificant among these imaging methods due to a limited number of lesions. Hence, we analyzed each lesion’s width parameter pairwise with the US using the Cleveland dot plot. The width difference between US with other imaging methods was considered significant (marked red rectangle; Fig. 9) if the difference between pairs is >3  mm. Only two lesions’ widths in R-value segmentation showed a significant difference. The width of R-value segmented lesions is nearly identical in most US-R-value pairs [Fig. 9(d)].

## 4 Conclusion

In this study, a smartphone-coupled handheld intraoral probe was used to image PpIX FL before and after PDT in a small cohort of patients who were undergoing PDT treatment for early stage oral cancer. In post-PDT images, the PpIX bleached area confirmed the area of therapeutic (∼635  nm) light delivery. The histopathological findings from PDT-treated tissues confirmed that the necrosis had reached a depth of 5 mm, which is reasonably consistent with expectations for light penetration at this wavelength.^23^^,^^60^^,^^61^ The 14 treated lesions showed a post-ALA R-value range of 1.65 to 3.11, whereas only one lesion with a low R-value of 1.75 showed post-PDT recurrence during follow-up (see Table S1 in the Supplementary Material). This patient with incomplete tumor response had moderately differentiated squamous cell carcinoma (mod. diff. SCC) compared to successful ones with well-differentiated squamous cell carcinoma (well. diff. SCC). The successfully treated patients showed no residual diseases during 50 weeks of median follow-up and among 14 lesions 13 showed the post-PDT complete tumor response. However, it is important to note this was a pilot phase evaluation of imaging hardware being used in a new clinical context and image data will need to be gathered from a much larger patient cohort before conclusions about correlation with imaging and later outcomes can be substantiated. Future implementation of this hardware should also incorporate measurement of tissue optical properties, which are expected to change during PDT and could account for differences in fluorophore absorption, which are otherwise conflated with changes in subsequent FL emission. While this could be important to more robust interpretation of tissue changes during auto-FL, the overall agreement in lesion segmentation with US indicates that this hardware works well for its primary intended purpose as a handheld tool for helping the clinician demarcate lesion size and position prior to PDT light delivery.

Overall the smartphone-coupled intraoral device successfully integrated features of existing auto-FL imaging devices^62^^–^^66^ while achieving the intended suitability for remote and rural healthcare settings. (1) It is portable, lightweight, durable, sustainable, and affordable. (2) It runs on battery power making it suitable for locations with limited infrastructure. (3) The device is connected to a cloud-based web application that automatically links up with the local network, making remote diagnosis quick and easy. (4) The device is user-friendly and can be operated by local community workers with minimal training. (5) The previously reported capabilities for remote diagnosis and mobile deep learning lesion classification can enable a system for providing health workers with triage instructions to guide them. The potential for acceptance of this technology overall as a low-cost, portable, and capable theranostic cancer technology for global health is supported by the wide availability of smartphone in global health settings.^67^ In the future, large-scale studies will be required to validate and calibrate the R-value ratiometric imaging during the monitoring and guidance of PDT. This approach could potentially discriminate between low to severe dysplasia before developing into CIS. It is also worth noting that implementation of this optical approach could be combined with US for assessment of the depth of invasion. For pre-malignant dysplasia, it is reasonable to assume there is no invasion but for invasive tumors, US would play an important role to establish depth of invasion. With appropriate consideration of these factors and if supported by larger scale studies, oral lesions could be screened and treated simultaneously in remote rural clinical settings with the integration of PDT light delivery and intraoral imaging in the same hardware.

## Code, Data, and Materials Availability

Details regarding methods and materials are provided in the Supplementary Material.
