# Supplementary material for: Clinical assessment of a low-cost, hand-held, smartphone-attached intraoral imaging probe for 5-aminolevulinic acid photodynamic therapy monitoring and guidance
Source: J Biomed Opt. 2023 Jul 21;28(8):082809. doi: 10.1117/1.JBO.28.8.082809 (PMC10362156; doi:10.1117/1.JBO.28.8.082809)

Supplementary Material

Clinical assessment of a low-cost, hand-held, smartphone-attached intraoral imaging probe for ALA PDT monitoring and guidance

Shakir Khan PhD[a, b, d], Bofan Song PhD[c], Srivalleesha Mallidi PhD[b], Shaobai Li PhD[c], Hui Liu PhD[a], M. A. Bilal Hussain MSc[d], Shaista Siddiqui FRCR[e], Amjad P. Khan PhD[b], Kafil Akhtar MD[g], Shahid Ali Siddiqui MD[d], Syed Abrar Hasan MS[h], Colin Hopper FRCS(Ed)[f], Stephen G. Bown FRCP[f], Rongguang Liang PhD[c], Tayyaba Hasan PhD*[b, i,] and Jonathan P. Celli PhD*[a]

[a]University of Massachusetts at Boston, Boston, Massachusetts, United States.
[b]Massachusetts General Hospital and Harvard Medical School, Boston, Massachusetts, United States.
[c]The University of Arizona, Wyant College of Optical Sciences, Tucson, Arizona, United States.
[d]Aligarh Muslim University, Jawaharlal Nehru Medical College, Department of Radiotherapy, Aligarh, India.
[e]Aligarh Muslim University, Jawaharlal Nehru Medical College, Department of Radiodiagnosis, Aligarh, India.
[f]University College London, London, England, United Kingdom.
[g]Aligarh Muslim University, Jawaharlal Nehru Medical College, Department of Pathology, Aligarh, India.
[h]Aligarh Muslim University, Jawaharlal Nehru Medical College, Department of Otorhinolaryngology (E.N.T.), Aligarh, India.
[i]Division of Health Sciences and Technology, Harvard University and Massachusetts Institute of Technology, Cambridge, MA, USA.

* Corresponding authors: Tayyaba Hasan PhD, Wellman Center for Photomedicine, Massachusetts General Hospital, Harvard Medical School, Boston MA 02114, E-mail: thasan@mgh.harvard.edu, Jonathan P Celli PhD, Physics Department, University of Massachusetts at Boston, Boston MA 02125 -3393, E-mail: Jonathan.celli@umb.edu

Table of contents:

S.N.	Supplementary Contents	P. N.
**Table S1**	Pre- and post-PDT histology impression and post-ALA lesions' R-value	**2**
**S1**	Box plot of paired, red- and green-channel fluorescence intensity (lesion site) at pre-, post-ALA, and post-PDT.	**3**
**S2**	12 patients lesion images and segmentation data at Pre-PDT, post-ALA, and post-PDT.	**4-15**
**S3**	Python image processing methods	**16**

**Table S1: Pre- and post-PDT histology impression and post-ALA lesions' *R-value***				
**Lesion ID**	Pre-PDT H&E (OSCC type)	Pre-PDT Dysplasia status	Post-ALA *Lesion site avg. R-value*	Post-PDT follow-ups status
**p11**	Mod. diff. SCC	Mild and marked dysplasia	2.29	Success/NED
**p12**	Mod. diff. SCC	Mild and marked dysplasia	2.48	Success/NED
**p13**	**Well. diff. SCC**	Mild and marked dysplasia	1.75	**Failure/recurrence**
**p14bm**	Mod. diff. SCC	dysplasia	2.85	Success/NED
**p14l**	Mod. diff. SCC	dysplasia	3.11	Success/NED
**p23**	Mod. diff. SCC	dysplasia	1.89	Success/NED
**p24**	Mod. diff. SCC	dysplasia	2.08	Success/NED
**p25**	Mod. diff. SCC	dysplasia	1.82	Success/NED
**p26lb**	Mod. diff. SCC	dysplastic stratified epithelium	2.04	Success/NED
**p26rb**	Mod. diff. SCC	dysplastic stratified epithelium	2.03	Success/NED
**p27**	Mod. diff. SCC	dysplasia	2.14	Success/NED
**p28**	Mod. diff. SCC	dysplastic stratified epithelium	1.93	Success/NED
**p29**	Mod. diff. SCC	mild dysplasia	1.65	Success/NED
**p30**	Mod. diff. SCC	Moderately + severe dysplasia	1.87	Success/NED
**NED: no evidence of disease, OSCC: Oral squamous cell carcinoma**				

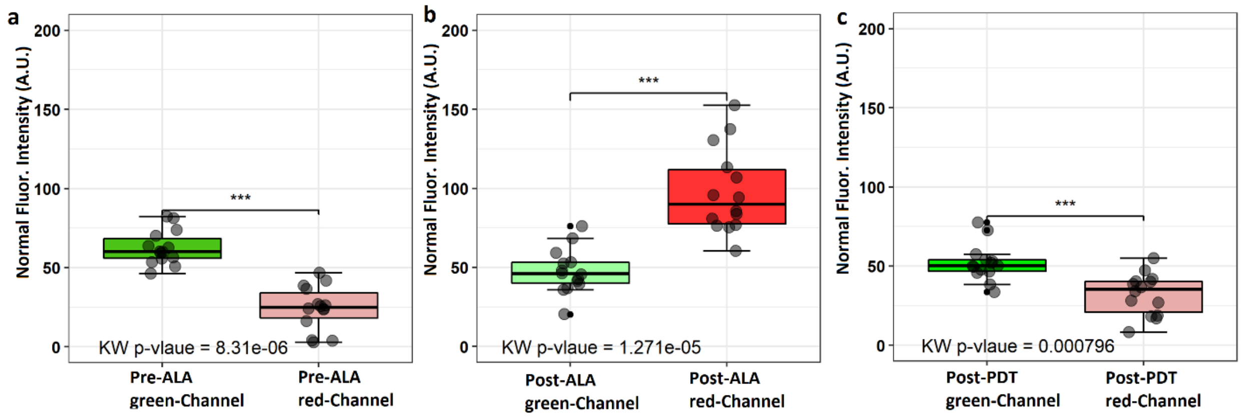

Fig. S1: Box plot of paired, red- and green-channel fluorescence intensity (lesion site) at pre-, post-ALA, and post-PDT.

S2: 12 patients lesion images and segmentation data at Pre-PDT, post-ALA and post-PDT timepoint.

Patient 11:

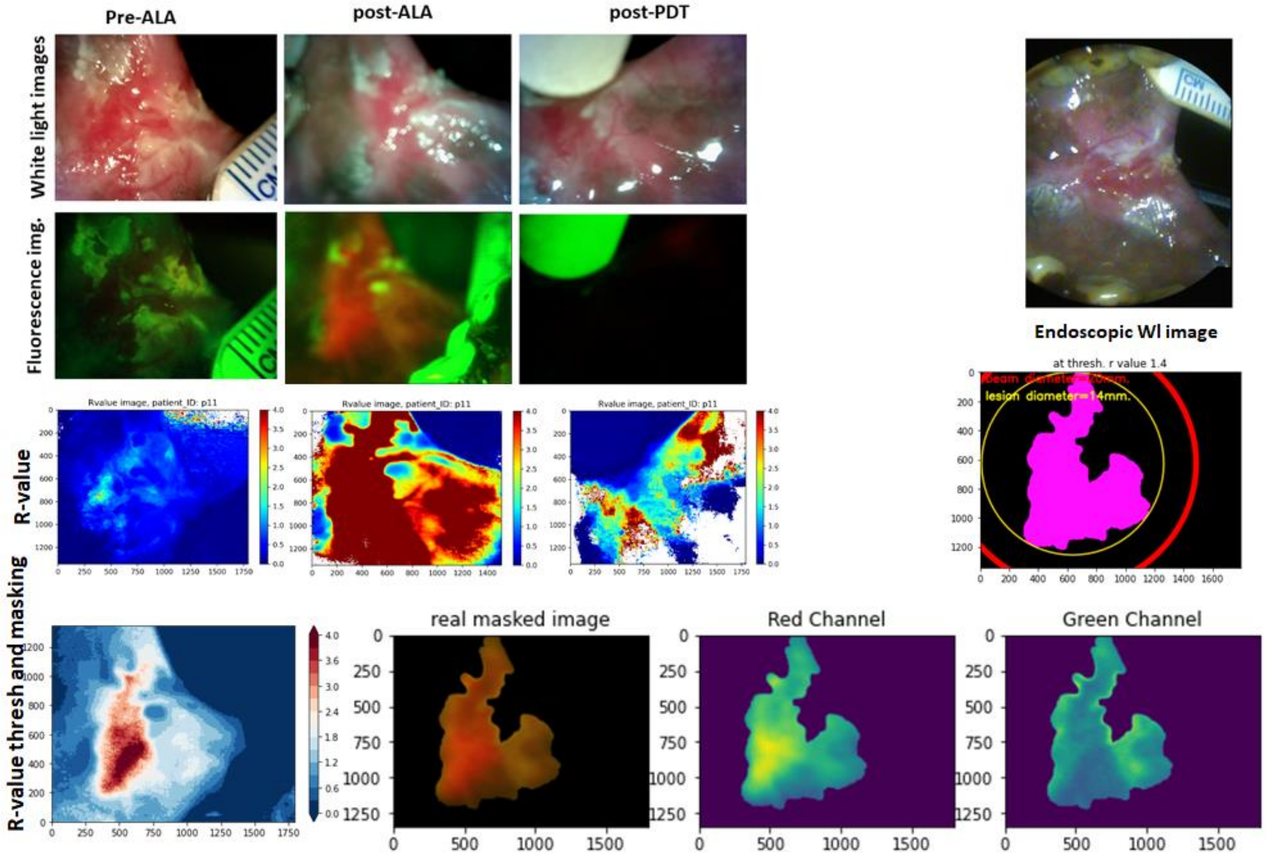

Patient 12:

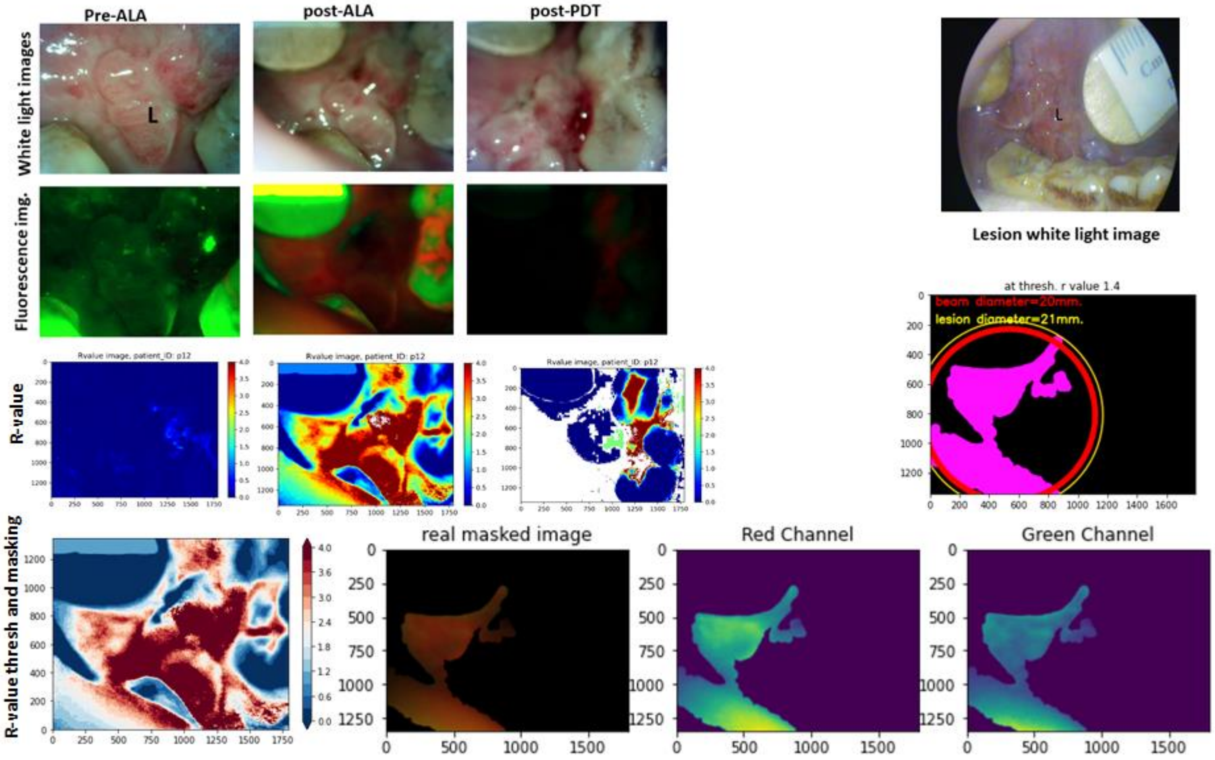
Pre-ALA
post-ALA
post-PDT
White light images
L
Fluorescence img.
R-value
Rvalue image, patient_ID: p12
Rvalue image, patient_ID: p12
Rvalue image, patient_ID: p12
R-value thresh and masking
real masked image
Red Channel
Green Channel
Lesion white light image
at thresh. r value 1.4
beam diameter=20mm.
lesion diameter=21mm.

Patient 13:

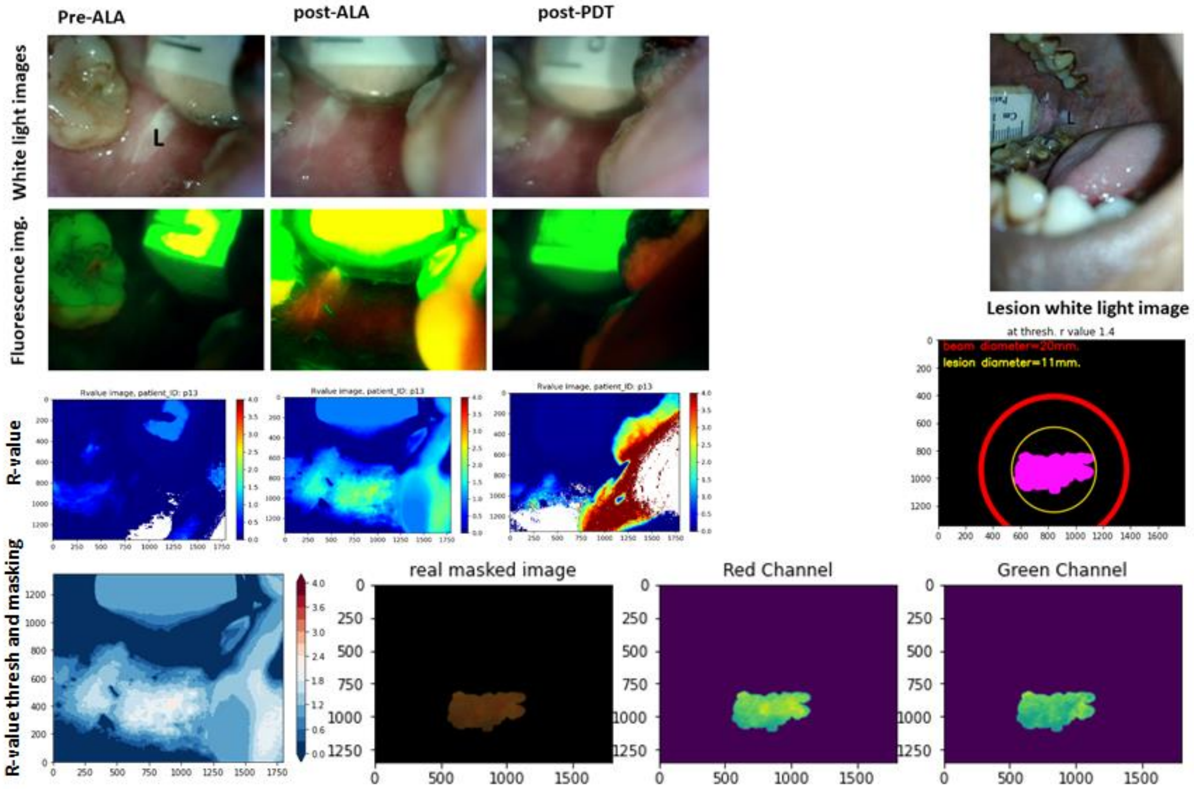
Pre-ALA
post-ALA
post-PDT
White light images
Fluorescence img.
R-value
R-value thresh and masking
Lesion white light image
at thresh. r value 1.4
beam diameter=20mm.
lesion diameter=11mm.
real masked image
Red Channel
Green Channel

Patient 14:

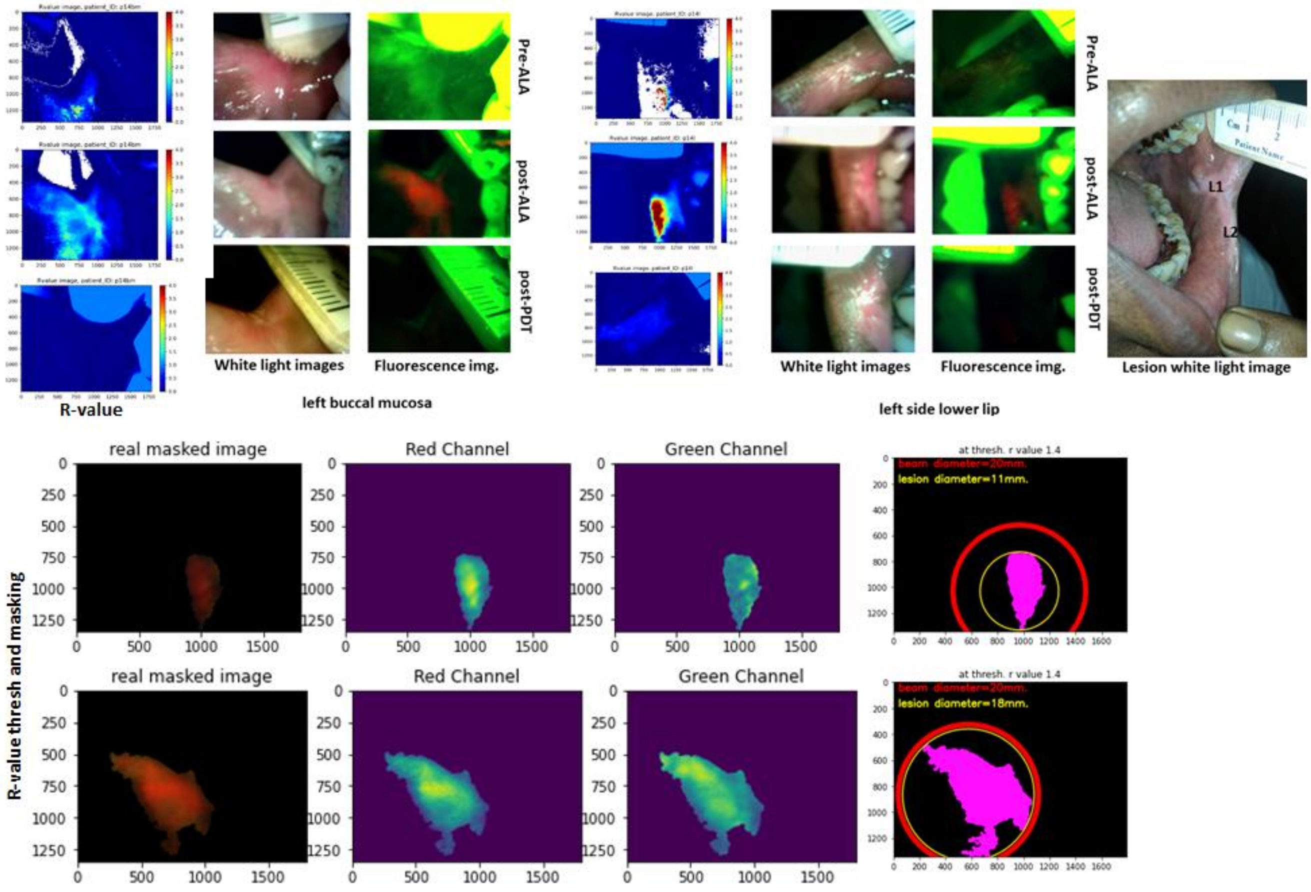
Pre-ALA
post-ALA
post-PDT
White light images
Fluorescence img.
R-value
left buccal mucosa
Pre-ALA
post-ALA
post-PDT
White light images
Fluorescence img.
Lesion white light image
L1
L2
left side lower lip
R-value thresh and masking
real masked image
Red Channel
Green Channel
at thresh. r value 1.4
beam diameter=20mm.
lesion diameter=11mm.
real masked image
Red Channel
Green Channel
at thresh. r value 1.4
beam diameter=20mm.
lesion diameter=18mm.

Patient 23:

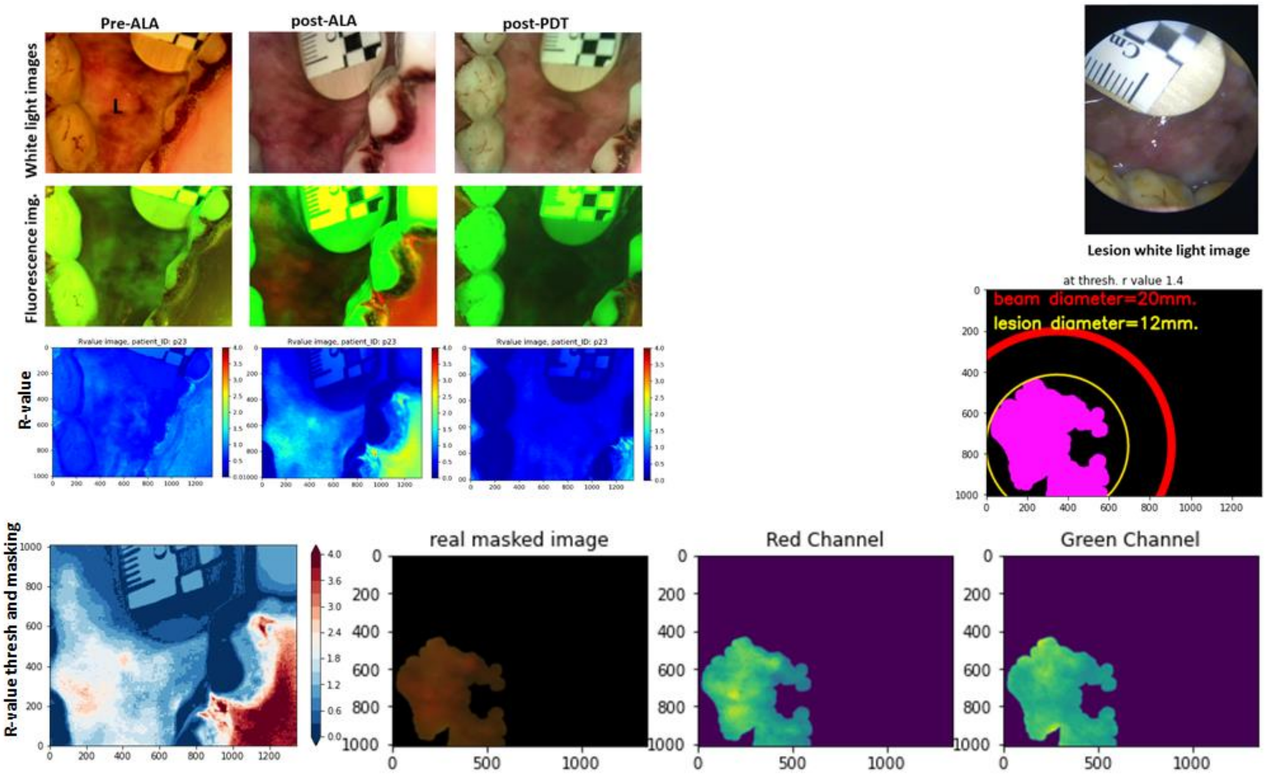
Pre-ALA
post-ALA
post-PDT
White light images
L
Fluorescence img.
R-value
Rvalue image, patient_ID: p23
Lesion white light image
at thresh. r value 1.4
beam diameter=20mm.
lesion diameter=12mm.
R-value thresh and masking
real masked image
Red Channel
Green Channel

Patient 24:

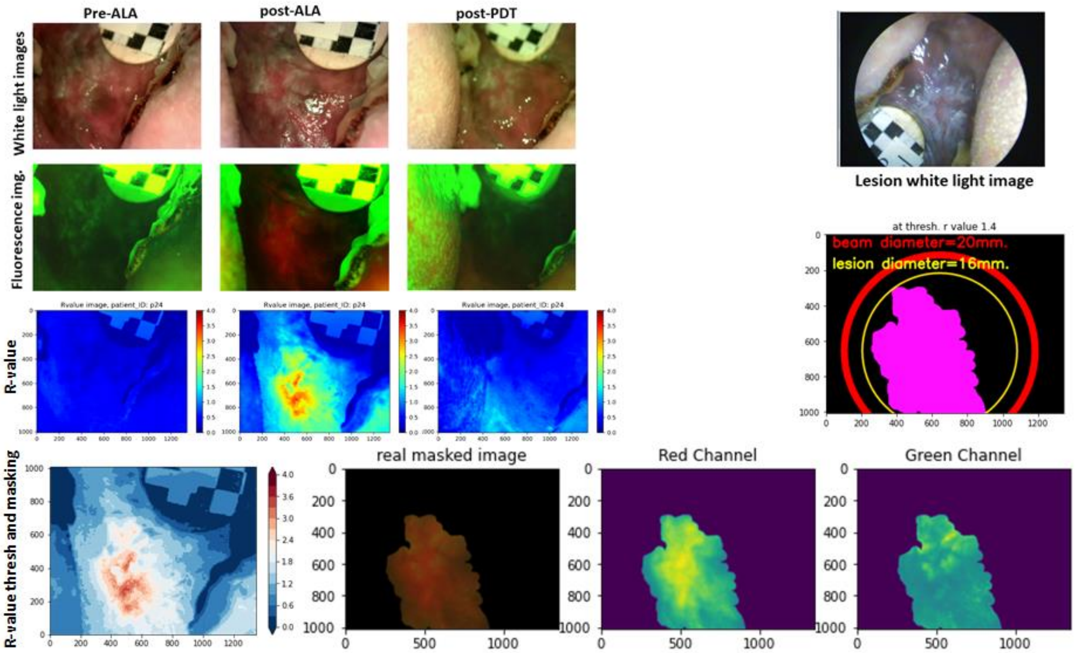

Pre-ALA
post-ALA
post-PDT
White light images
Fluorescence img.
R-value
R-value thresh and masking
Lesion white light image
at thresh. r value 1.4
beam diameter=20mm.
lesion diameter=16mm.
real masked image
Red Channel
Green Channel

Patient 25:

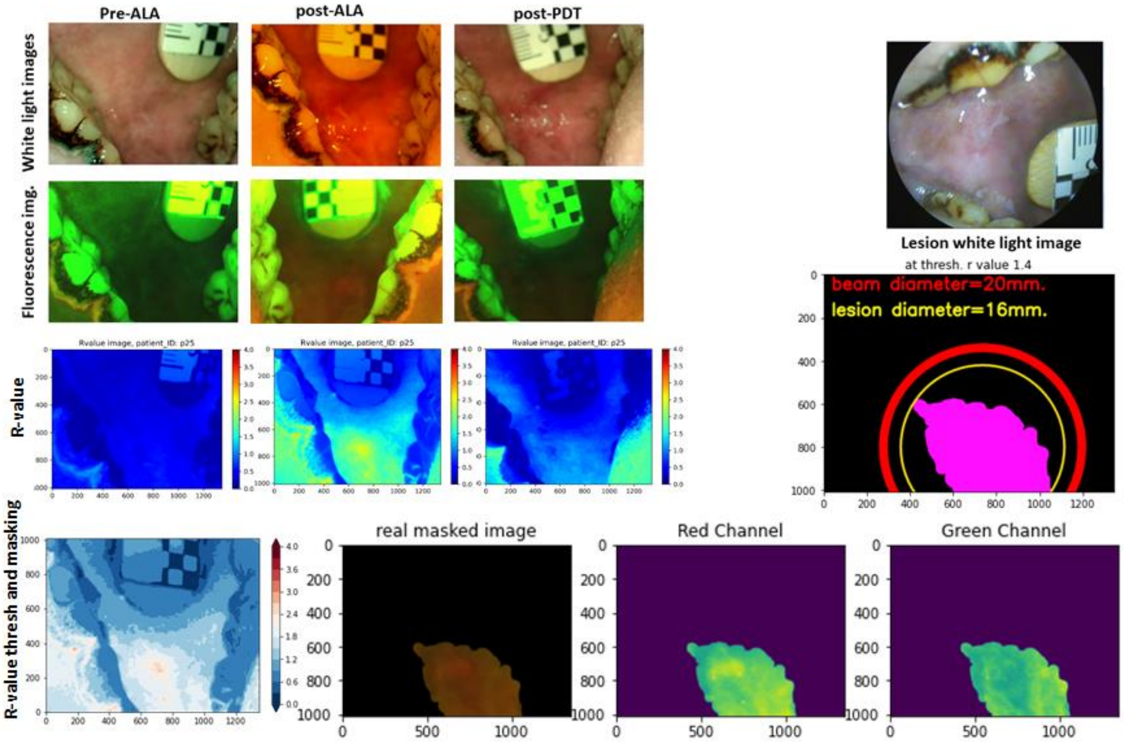
Pre-ALA
post-ALA
post-PDT
White light images
Fluorescence img.
R-value
R-value thresh and masking
Lesion white light image
at thresh. r value 1.4
beam diameter=20mm.
lesion diameter=16mm.
real masked image
Red Channel
Green Channel

Patient 26:

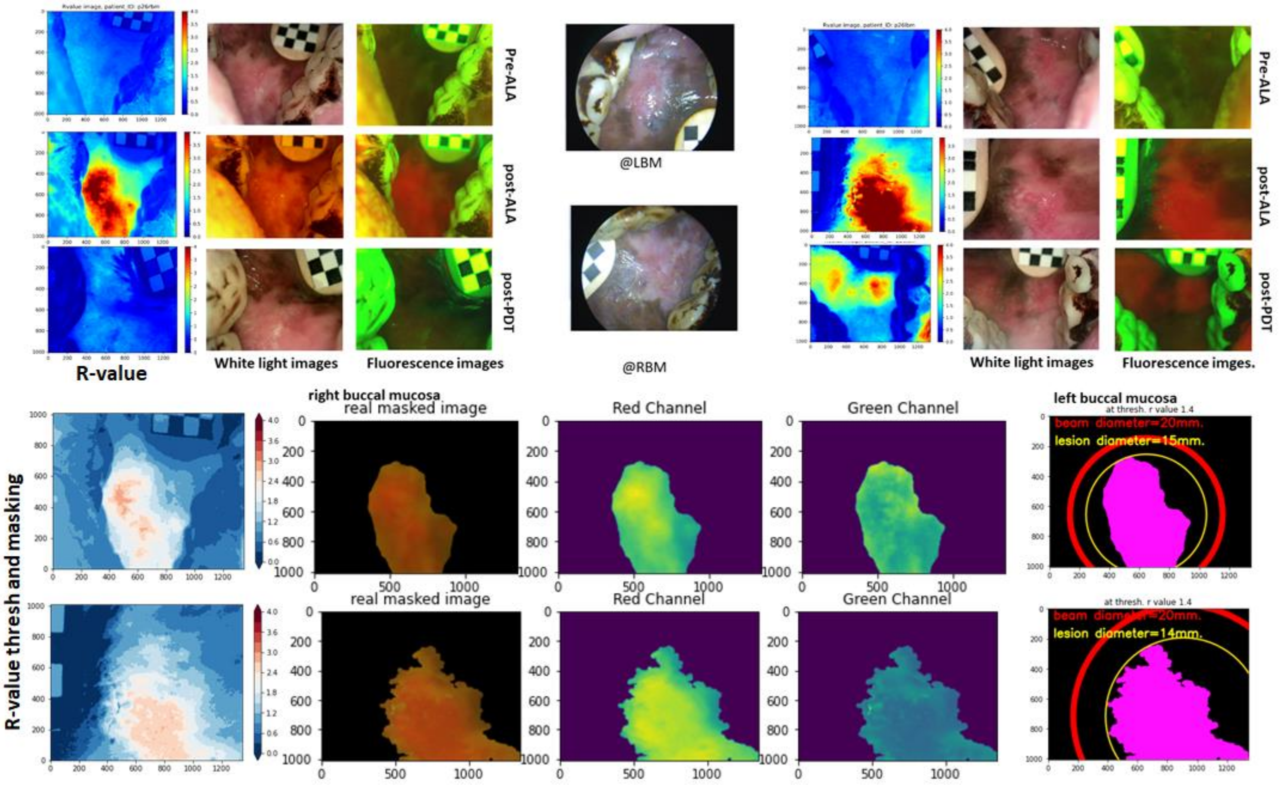

Pre-ALA
post-ALA
post-PDT
R-value
White light images
Fluorescence images
@LBM
@RBM
White light images
Fluorescence imges.
R-value thresh and masking
right buccal mucosa
real masked image
Red Channel
Green Channel
left buccal mucosa
at thresh. r value 1.4
beam diameter=20mm.
lesion diameter=15mm.
real masked image
Red Channel
Green Channel
at thresh. r value 1.4
beam diameter=20mm.
lesion diameter=14mm.

Patient 27:

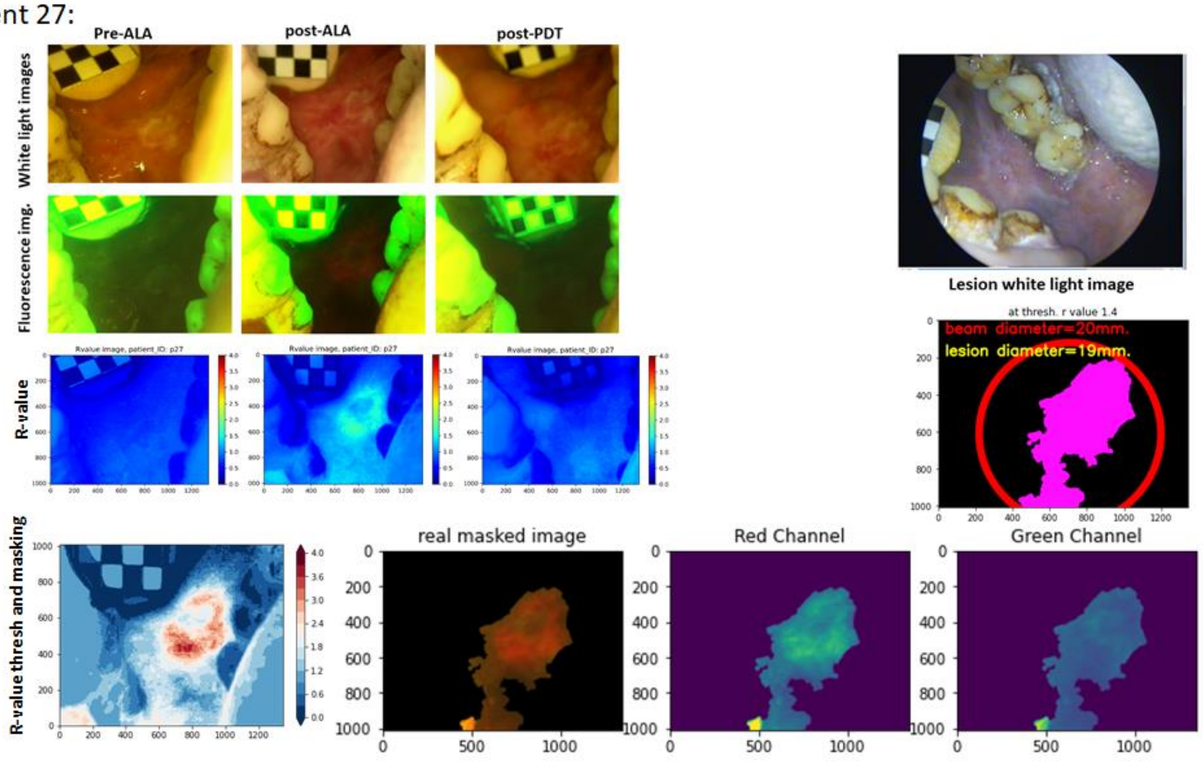

Pre-ALA
post-ALA
post-PDT
White light images
Fluorescence img.
R-value
Rvalue image, patient_ID: p27
Rvalue image, patient_ID: p27
Rvalue image, patient_ID: p27
Lesion white light image
at thresh. r value 1.4
beam diameter=20mm.
lesion diameter=19mm.
R-value thresh and masking
real masked image
Red Channel
Green Channel

Patient 28:

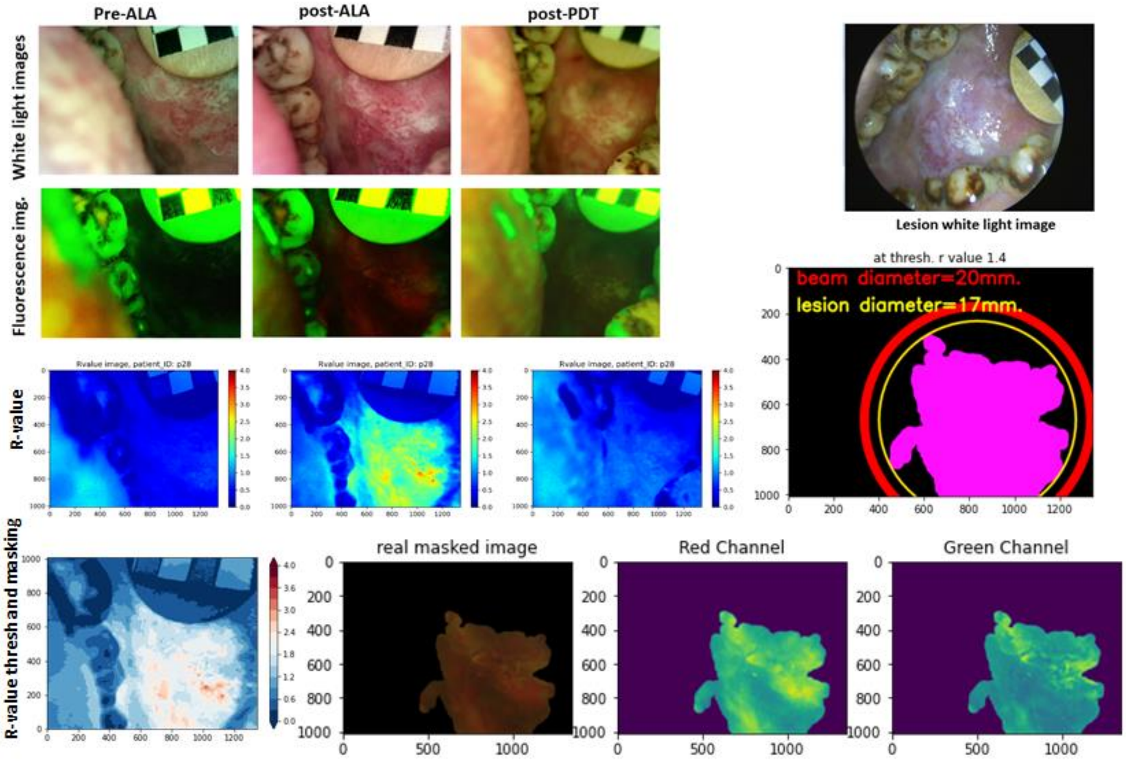

Pre-ALA
post-ALA
post-PDT
White light images
Fluorescence img.
R-value
R-value thresh and masking
Lesion white light image
at thresh. r value 1.4
beam diameter=20mm.
lesion diameter=17mm.
real masked image
Red Channel
Green Channel

Patient 29:

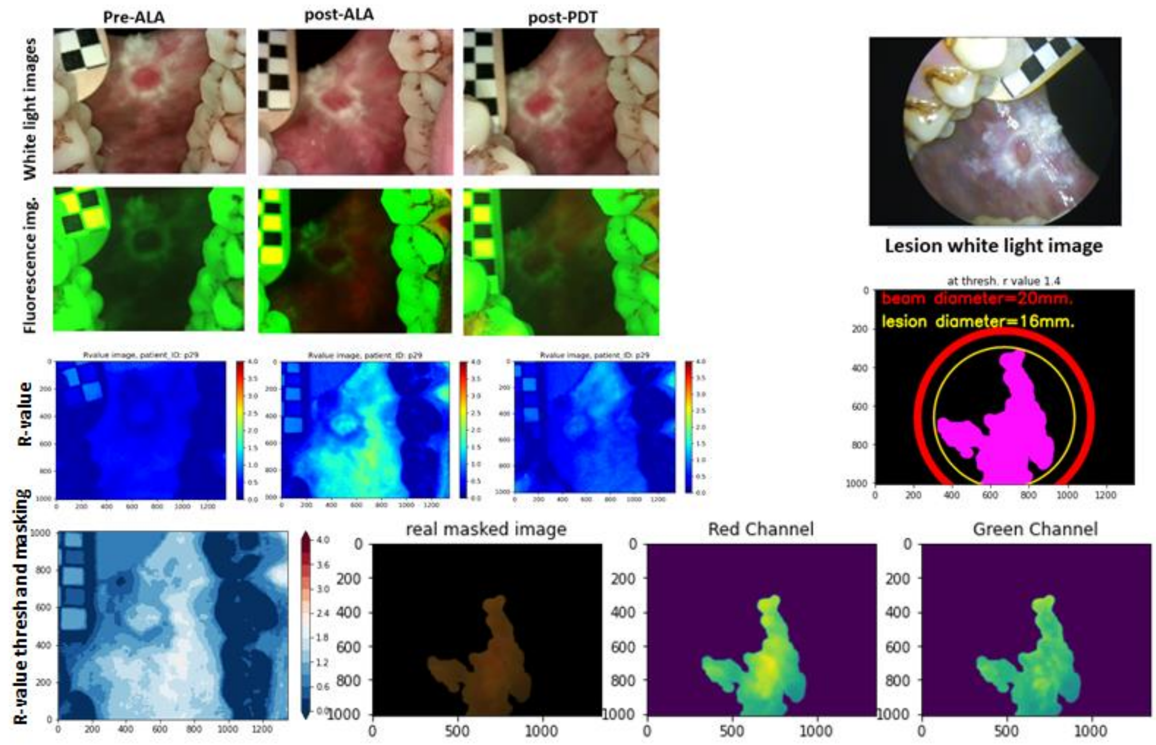

Pre-ALA
post-ALA
post-PDT
White light images
Fluorescence img.
R-value
R-value thresh and masking
Lesion white light image
at thresh. r value 1.4
beam diameter=20mm.
lesion diameter=16mm.
real masked image
Red Channel
Green Channel

Patient 30:

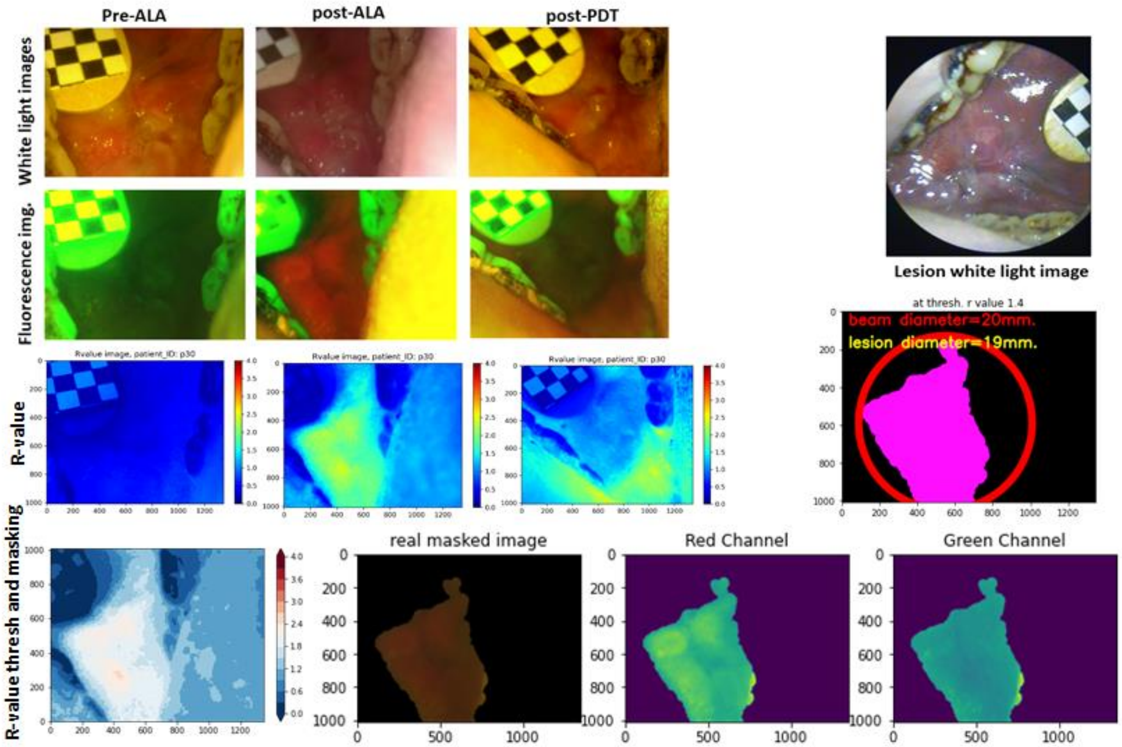
Pre-ALA
post-ALA
post-PDT
White light images
Fluorescence img.
R-value
Rvalue image, patient_ID: p30
R-value thresh and masking
Lesion white light image
at thresh. r value 1.4
beam diameter=20mm.
lesion diameter=19mm.
real masked image
Red Channel
Green Channel

S3: Python image processing methods:

Image processing methods for Intra-Oral Probe mediated Clinical PDT images

January 20, 2023

0.1 Flow-chart

[4]:
```
import import_ipynb
from flow_chart import *
chart()
```

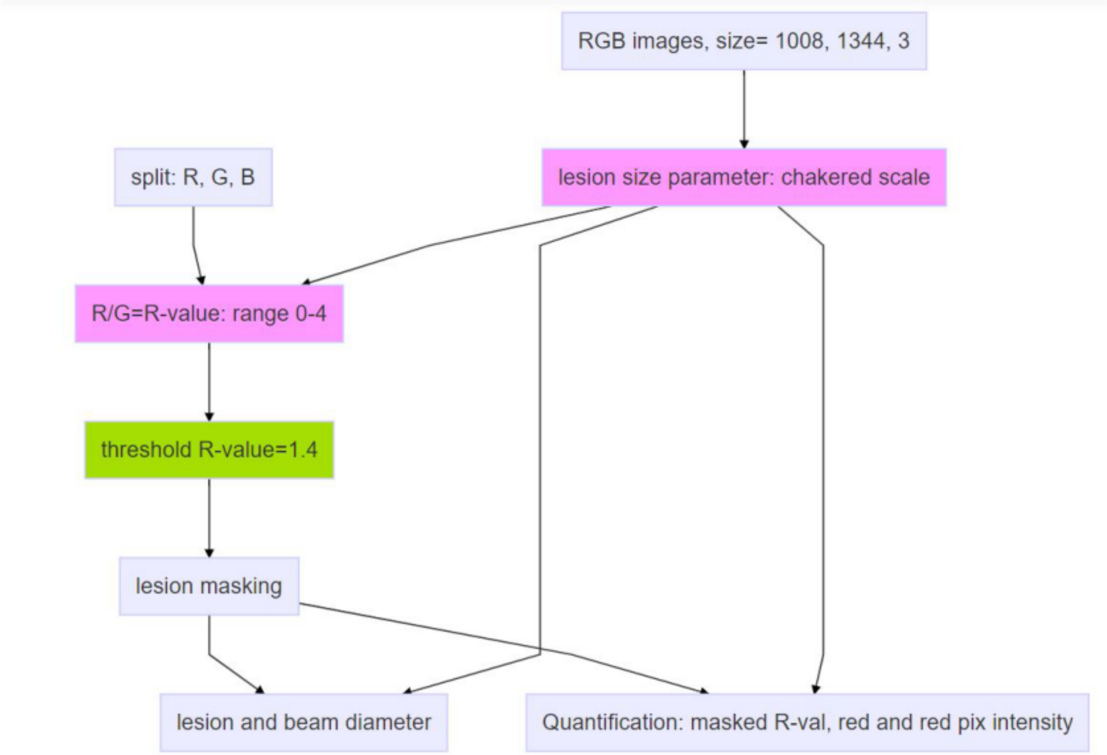

0.2 Set the images folder path and working directory

[2]:
```
import os
cwd = os.getcwd()
#-------------- Print the current working directory-------------------------#
```

```
print("Current working directory: {0}".format(cwd))
path='C:/Users/su623/Desktop/liang_JBO_manuscript/liang_image_processing/
↪postALA' #set the images working directory
os.chdir(path)
cwd = os.getcwd()
#---------------- Print the image working directory-------------------------#
print("Current working directory: {0}".format(cwd))
```

```
Current working directory: C:\Users\su623
Current working directory:
C:\Users\su623\Desktop\liang_JBO_manuscript\liang_image_processing\postALA
```

0.3 Libraries for image processing steps

[5]:
```
import numpy as np #numPy is a Python library used for working with arrays␣
↪(image arrays).
import cv2 # OpenCV packages for image processing.
import matplotlib.pyplot as plt #matplotlib.pyplot is a collection of functions␣
↪to unable the matplotlib work like MATLAB.
from matplotlib import colors #a list of the named colors.
from matplotlib import cm # for colormaps.
import matplotlib.colors as colors # a list of the named colors for map␣
↪plotting.
from matplotlib.pyplot import pcolormesh #Create a pseudocolor plot with a␣
↪non-regular rectangular grid.
import glob# The glob. iglob() works exactly the same as the glob() method.
import warnings # to stop the warnings in the executed cell.
import sympy
```

0.4 Split the RGB images into red, green, blue channels :

0.4.1 Load the (1) pre-PDT (2) post-ALA (3) post-PDT .jpg images

0.4.2 Dimension of each image: 1344x1792 pixel dimension and 8-bit depth.

0.5 Post-ALA RGB image

[6]:
```
#---------------------- original RGB image---------------------------------#
real_image='C:/Users/su623/Desktop/liang_JBO_manuscript/liang_image_processing/
↪postALA/p24.jpg'
real_img = cv2.imread(real_image)
print(real_img.shape)
real_img = cv2.cvtColor(real_img, cv2.COLOR_BGR2RGB)

plt.imshow(real_img, origin='lower') # original image RGB, showing PpIX␣
↪fluorescence (post-ALA)
```

```
(1008, 1344, 3)
```

[6]: <matplotlib.image.AxesImage at 0x29a6942e130>

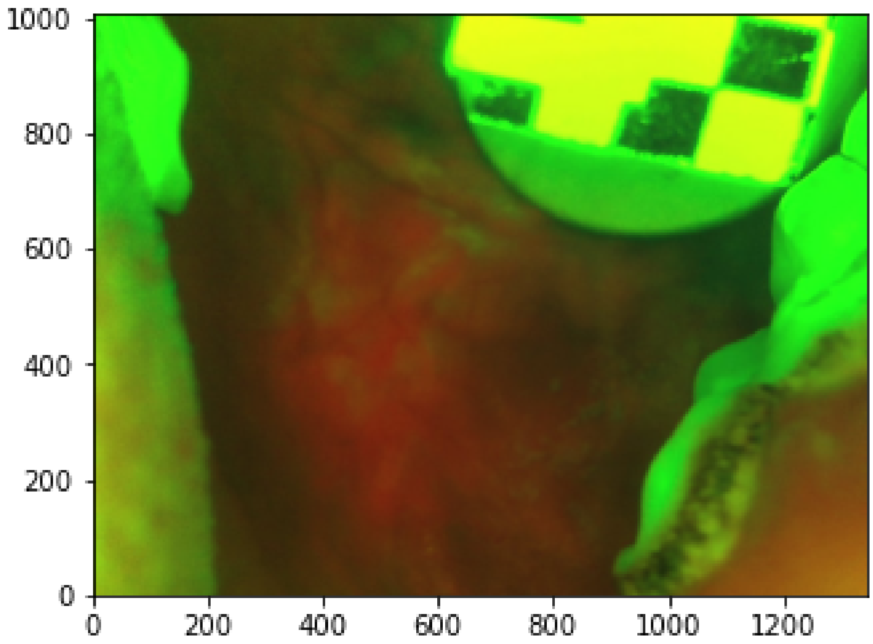

0.6 Get the R, G, B split channel images

[7]:
```
real_img = cv2.imread(real_image)
B, G, R = cv2.split(real_img)
#------------show the splitted image (pseudo color gray images, B, G,␣
↪R)---------#
fig, ax = plt.subplots(1,3, figsize=(14,14) )
fig.tight_layout()
#--------------create␣
↪subplots-----------------------------------------------------#
ax[ 0].imshow(R, origin='lower')
ax[0].set_title('Red channel')
ax[ 1].imshow(G, origin='lower')
ax[1].set_title('Green channel')
ax[ 2].imshow(B, origin='lower')
ax[2].set_title('Blue channel')
#plt.savefig('preALA_.jpg', dpi=300)
```

[7]: Text(0.5, 1.0, 'Blue channel')

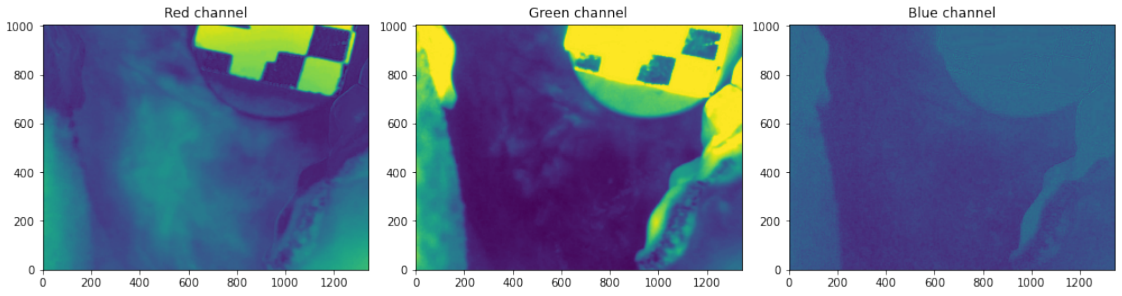

0.7 Ratio values: ratio=R/G (pseudo channel image based ratio/R-value).

[62]:
```
Rval=sympy.Symbol('R_value(0-4)')
i = sympy.Symbol('i')

Ired=sympy.Symbol('I_red@(x, y)')
Igreen=sympy.Symbol('I_green@(x,y)')
Iblue=sympy.Symbol('I_blue@(x,y)')
IaryRed=sympy.Symbol('I_red@(x, y)[1-255]')
IaryGreen=sympy.Symbol('I_green@(x, y)[1-255]')
sumIred=sympy.Sum(Ired, (i, ('x', 'y'), ))
sumIgreen=sympy.Sum(Igreen, (i, 1, 255))
sumIblue=sympy.Sum(Iblue, (i, 1, 255))
I_total=Ired + Igreen + Iblue
Itoal_intensity=sympy.Eq(sympy.Symbol('I_total(x, y)'), I_total  )
IredNorm=sympy.Symbol('I_redNormal.')
IgreenNorm=sympy.Symbol('I_greenNormal.')
Itotal1_255=sympy.Symbol('I_total(x,y)')
sympy.Eq(IredNorm, IaryRed/Itotal1_255)
sympy.Eq(IgreenNorm, IaryGreen/Itotal1_255)
rvalue=4*IredNorm/IgreenNorm
#sympy.init_printing(use_latex='png', fontsize='5pt')
Itoal_intensity
```

[62]:

$$I_{total(x,y)} = I_{blue@(x,y)} + I_{green@(x,y)} + I_{red@(x,y)}$$

[63]:
```
sympy.Eq(IredNorm, IaryRed/Itotal1_255)
```

[63]:

$$I_{redNormal.} = \frac{I_{red@(x,y)[1-255]}}{I_{total(x,y)}}$$

[64]:
```
sympy.Eq(IgreenNorm, IaryGreen/Itotal1_255)
```

[64]:

$$I_{greenNormal.} = \frac{I_{green@(x,y)[1-255]}}{I_{total(x,y)}}$$

[65]:
```
sympy.Eq(Rval, rvalue)
```

[65]:

$$R_{value(0-4)} = \frac{4I_{redNormal.}}{I_{greenNormal.}}$$

[96]:

```
#---------------get the r value or ratio and show the gray r value␣
↪image----------------#
ratio=R/G
ratio
plt.imshow(ratio, origin='lower', vmin=0, vmax=4)
warnings.filterwarnings('ignore') #
#ratio.shape
```

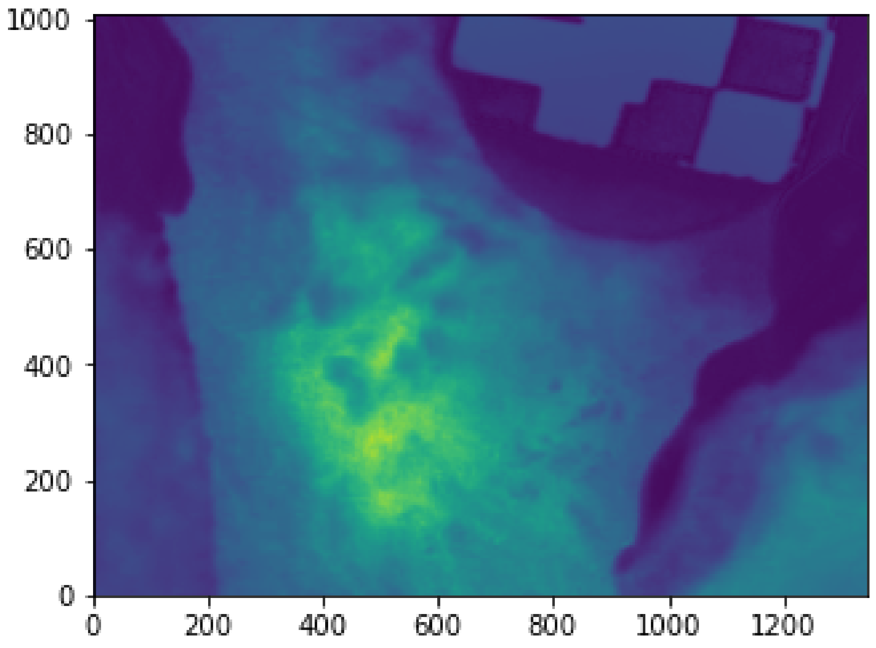

0.8 R-value range; 0 to 4 (0.3 steps)

[7]:

```
bounds = np.array([ 0, 0.3,  0.6, 0.9, 1.2, 1.5, 1.8, 2.1, 2.4, 2.7, 3.0, 3.3,␣
↪3.6, 3.9, 4.0 ]) #set the R_threshold-
#value range 0-4
norm = colors.BoundaryNorm(boundaries=bounds, ncolors=256)
pcm = pcolormesh(ratio, norm=norm, cmap='RdBu_r')
plt.colorbar(pcm,  extend='both', orientation='vertical')
```

[7]:

```
<matplotlib.colorbar.Colorbar at 0x1ee925cee20>
```

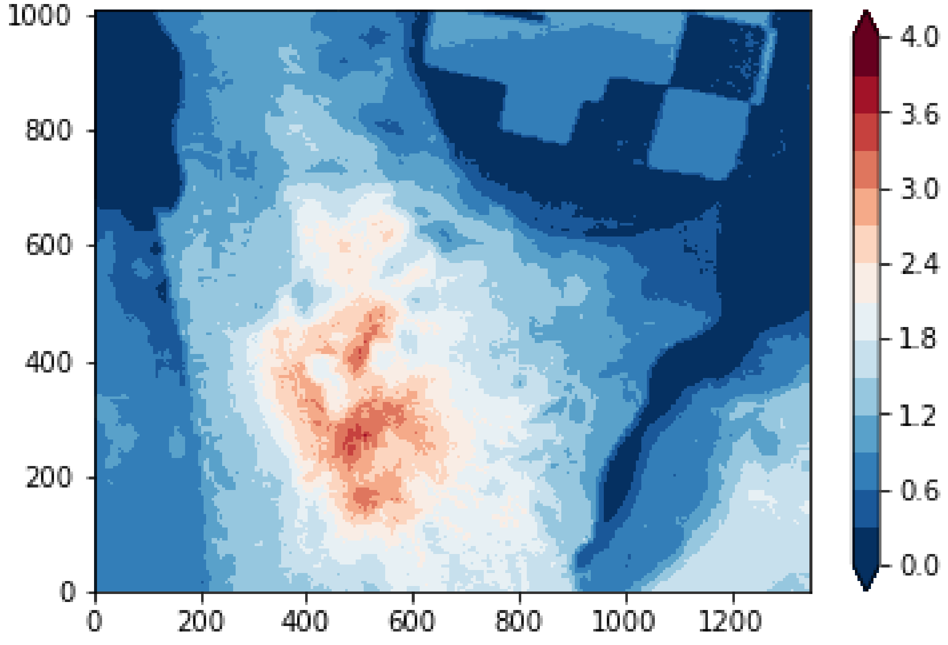

0.9 Set the threshold value 1.4, 1.8 for segmentation of lesion with and without margins.

0.9.1 Ex: The R value 1.4 (threshold value)

[8]:
```
#import matplotlib.colors as colors
#from matplotlib.pyplot import pcolormesh
bounds = np.array([ 0,  1.4, 1.8, 2.0, 2.6, 3.2, 3.8, 4]) #set the R_threshold␣
↪value 1.8 (for lesion -
# without margins)
norm = colors.BoundaryNorm(boundaries=bounds, ncolors=256)
pcm = pcolormesh(ratio, norm=norm, cmap='RdBu_r')
plt.colorbar(pcm,  extend='both', orientation='vertical')
```

[8]:
```
<matplotlib.colorbar.Colorbar at 0x1ee9267ea00>
```

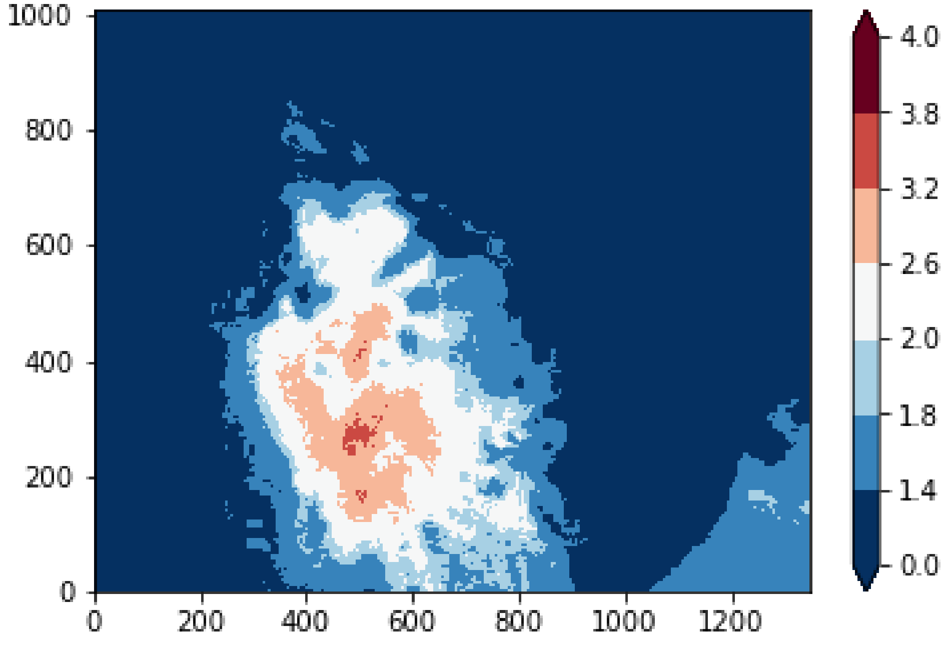

[9]:
```
#import pylab as plt
#from matplotlib import cm
#type(ratio.shape)
#ratio.shape
mask_ratio=ratio > 1.4 # masking of r vlaue with rvlaue threshold =1.8
cmap = cm.coolwarm
plt.imshow(mask_ratio, vmin=0, vmax=4, cmap=cmap, aspect='auto', origin='lower')
plt.colorbar()
```

[9]: <matplotlib.colorbar.Colorbar at 0x1ee928844c0>

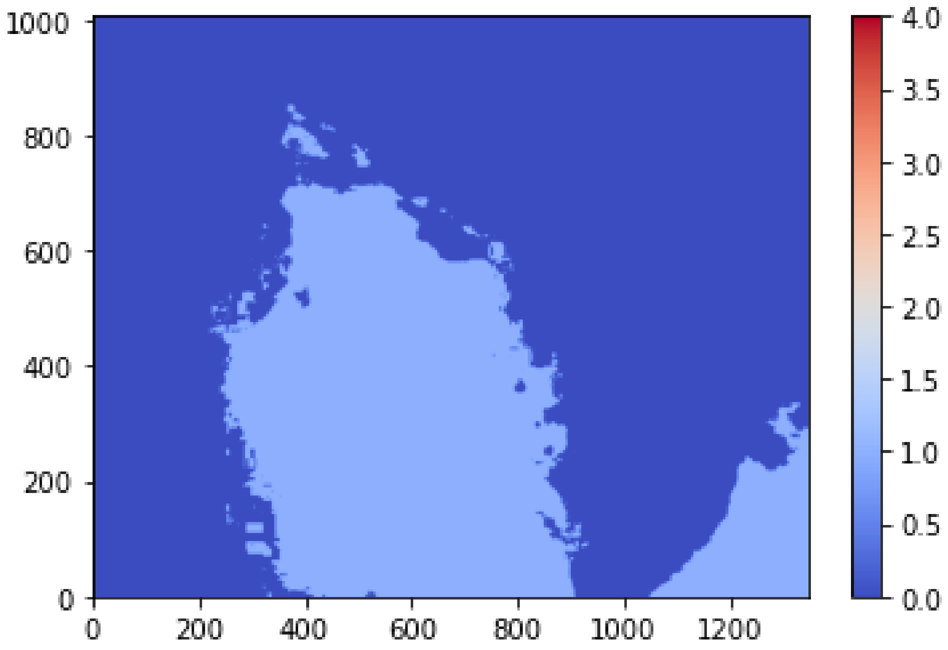

[10]:
```
cmap = colors.ListedColormap(['white', 'red'])
bounds=[0,1.4,4]
norm = colors.BoundaryNorm(bounds, cmap.N)
#---------tell imshow about color map so that only set colors are used-------#
img = plt.imshow(ratio, interpolation='bicubic', origin='lower',cmap=cmap,␣
 ↪norm=norm)
#----------make a color bar------------------------------------------------#
plt.colorbar(img, cmap=cmap, norm=norm, boundaries=bounds, ticks=[0, 1.4, 4]) #␣
 ↪set the R_threshold value 1.4 (for lesion -
# without margins)
# Threshold set to 1.4 R-values
warnings.filterwarnings('ignore') #ignore warning in the cell.
```

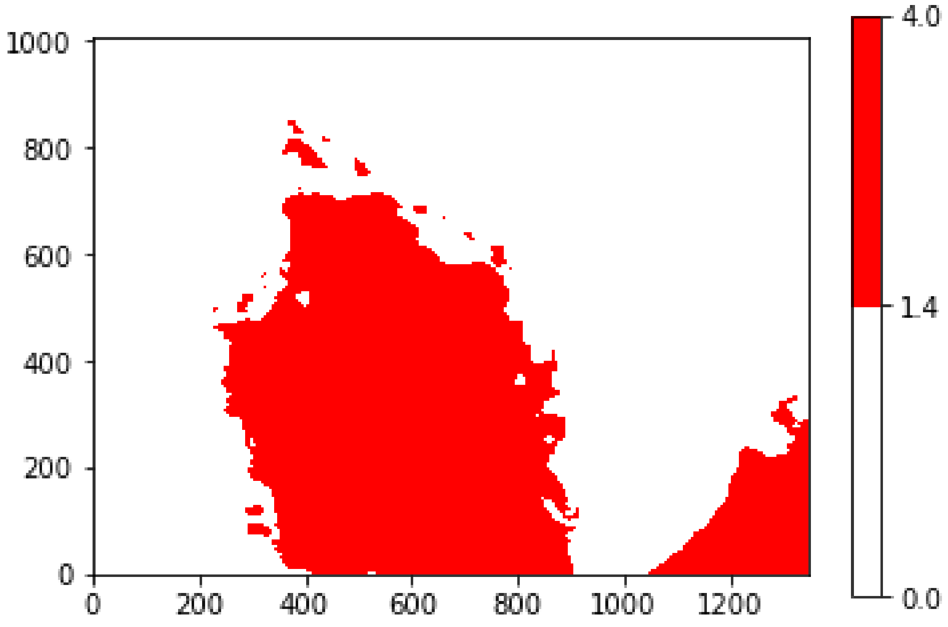

0.10 Get the size parameters of r image lesion using ref. scale (checkered scale)

[11]:
```
# original RGB image
real_image='C:/Users/su623/Desktop/liang_JBO_manuscript/liang_image_processing/
  ↪postALA/p24.jpg'
real_img = cv2.imread(real_image)
real_img1 = cv2.cvtColor(real_img, cv2.COLOR_BGR2RGB)

gray1 = cv2.cvtColor(real_img1, cv2.COLOR_BGR2GRAY)
thresh1 = cv2.threshold(gray1, 160, 255,
   cv2.THRESH_BINARY_INV)[1]

contours, hierarchy = cv2.findContours(thresh1.copy(), cv2.RETR_EXTERNAL,
   cv2.CHAIN_APPROX_SIMPLE)

for i, c in enumerate(contours):
   areaContour=cv2.contourArea(c)

def midpoint(ptA, ptB):
    return ((ptA[0] + ptB[0]) * 0.5, (ptA[1] + ptB[1]) * 0.5)

pix_per_mm=int(711/13)
real_imagecopy=real_img1.copy()
for i, c in enumerate(contours):
    areaContour=cv2.contourArea(c)
```

```
    if areaContour<2000 or 100000<areaContour:
        continue

    (x, y), (w, h), angle=cv2.minAreaRect(c)
    print(f'x and y vlaues are: {x, y}')
    print(f'width and height vlaues are: {w, h}')
    print(f'the angle is: {angle}')
    w_mm=w/pix_per_mm
    h_mm=h/pix_per_mm

    rect=cv2.minAreaRect(c)
    box=cv2.boxPoints(rect)
    box=np.int0(box)

    cv2.polylines(real_imagecopy, [box], True, (225, 0, 0), 10)
    cv2.putText(real_imagecopy, f'W: {round(w_mm, 1)}mm.',  (int(x-500),␣
↪int(y-80)), cv2.FONT_HERSHEY_PLAIN, 6, (0, 0, 255), 10)
    cv2.putText(real_imagecopy, f'H: {round(h_mm, 1)}mm.',  (int(x-500),␣
↪int(y-150)), cv2.FONT_HERSHEY_PLAIN, 6, (255, 0, 0), 10)
    (tl, tr, br, bl) = box
    #print(tl, tr, br, bl)
    (tltrX, tltrY) = midpoint(tl, tr)
    (blbrX, blbrY) = midpoint(bl, br)
        # compute the midpoint between the top-left and top-right points,
        # followed by the midpoint between the top-righ and bottom-right
    (tlblX, tlblY) = midpoint(tl, bl)
    (trbrX, trbrY) = midpoint(tr, br)
        # draw the midpoints on the image
    cv2.circle(real_imagecopy, (int(tltrX), int(tltrY)), 20, (255, 0, 0), -1)
    cv2.circle(real_imagecopy, (int(blbrX), int(blbrY)), 20, (255, 0, 0), -1)
    cv2.circle(real_imagecopy, (int(tlblX), int(tlblY)), 20, (255, 0, 0), -1)
    cv2.circle(real_imagecopy, (int(trbrX), int(trbrY)), 20, (255, 0, 0), -1)
    # draw lines between the midpoints
    cv2.line(real_imagecopy, (int(tltrX), int(tltrY)), (int(blbrX), int(blbrY)),
    (0, 0, 255), 4)
    cv2.line(real_imagecopy, (int(tlblX), int(tlblY)), (int(trbrX), int(trbrY)),
    (255, 0, 0), 4)

plt.imshow(real_imagecopy)
```

```
x and y vlaues are: (1175.0577392578125, 927.7885131835938)
width and height vlaues are: (151.20553588867188, 128.2599334716797)
the angle is: -11.309932708740234
```

[11]: <matplotlib.image.AxesImage at 0x1ee92b15f10>

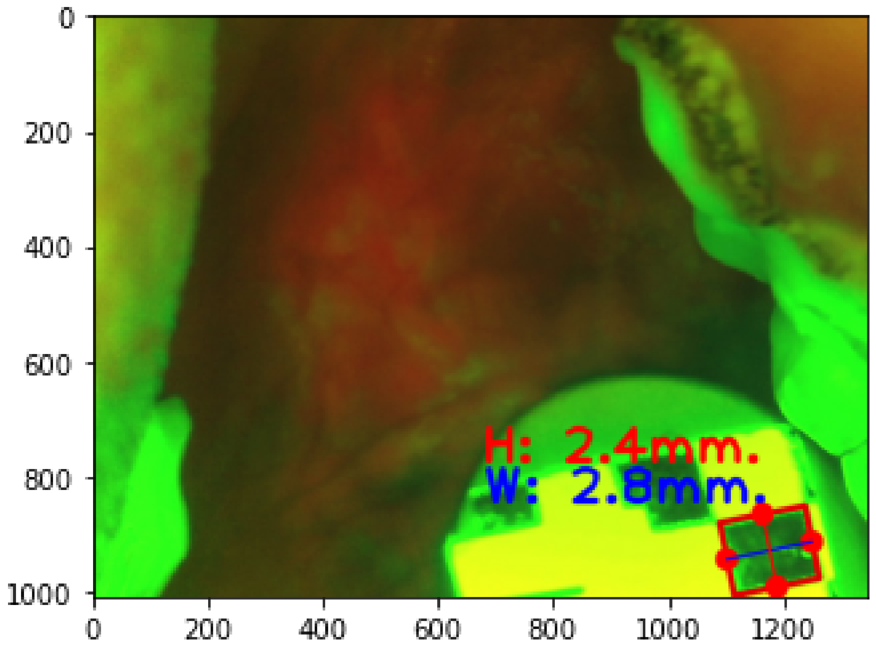

0.10.1 lesion diameter and superimpose beam diameter

[61]:
```
im = np.array((ratio * 255)/4, dtype = np.uint8) # .............
#imshow(im)
ret, thresh1 = cv2.threshold(im, 89, 255, cv2.THRESH_BINARY)
#plt.imshow(thresh1)

thresh2=thresh1.copy()

kernel = cv2.getStructuringElement(cv2.MORPH_ELLIPSE, (30,30))
opening = cv2.morphologyEx(thresh2, cv2.MORPH_OPEN, kernel, iterations=2)

contours, hierarchy = cv2.findContours(opening,cv2.RETR_TREE, cv2.
 ↪CHAIN_APPROX_NONE)

mask = np.zeros(real_img.shape, dtype=np.uint8)

if (i, areaContour) == max((i,cv2.contourArea(c)) for i,c in␣
 ↪enumerate(contours)):
    cv2.drawContours(mask,contours,i,(255,15,255),thickness=cv2.FILLED)

    (x,y),  radius = cv2.minEnclosingCircle(contours[i])
    cv2.circle(mask, (int(x), int(y)), int(radius), (255,225,0), 10)
    pix_per_mm=int(54.6)
    maximum_width=2*((radius/int(pix_per_mm)))
```

```
r_10=int(pix_per_mm*10)

R_=r_10-radius
new_radius=R_+ radius
cv2.circle(mask, (int(x), int(y)), int(new_radius), (255,0,0), 40)

beam_maximum_width=2*((new_radius/int(pix_per_mm)))

mask=cv2.flip(mask, 1)
mask = cv2.rotate(mask, cv2.ROTATE_180)
cv2.putText(mask, f'lesion diameter={int(maximum_width)}mm.', (int(50 -
↪15), int(200 - 10)),
          cv2.FONT_HERSHEY_SIMPLEX, 2.65, (255, 255,0), 10)
cv2.putText(mask, f'beam diameter={int(beam_maximum_width)}mm.', (int(50 -
↪15), int(80 - 10)),
              cv2.FONT_HERSHEY_SIMPLEX, 2.65, (255, 0), 10)

plt.imshow(mask)
plt.title('at thresh. R-value 1.4')
```

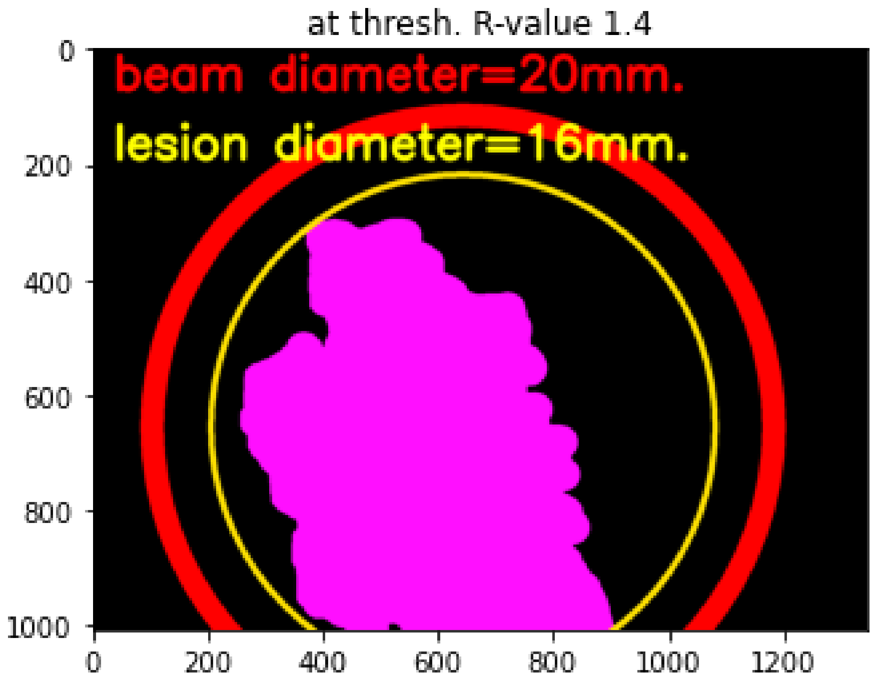

0.11 PDT light Applicatior and beam area upon lesion surface

[73]:
```
eq_image='C:/Users/su623/Desktop/liang_JBO_manuscript/figures/prop_beam.jpg'
eq_img = cv2.imread(eq_image)
eq_img = cv2.cvtColor(eq_img, cv2.COLOR_BGR2RGB)
plt.figure(figsize = (7,7))
plt.imshow(eq_img)

plt.axis('off')
```

[73]: (-0.5, 322.5, 275.5, -0.5)

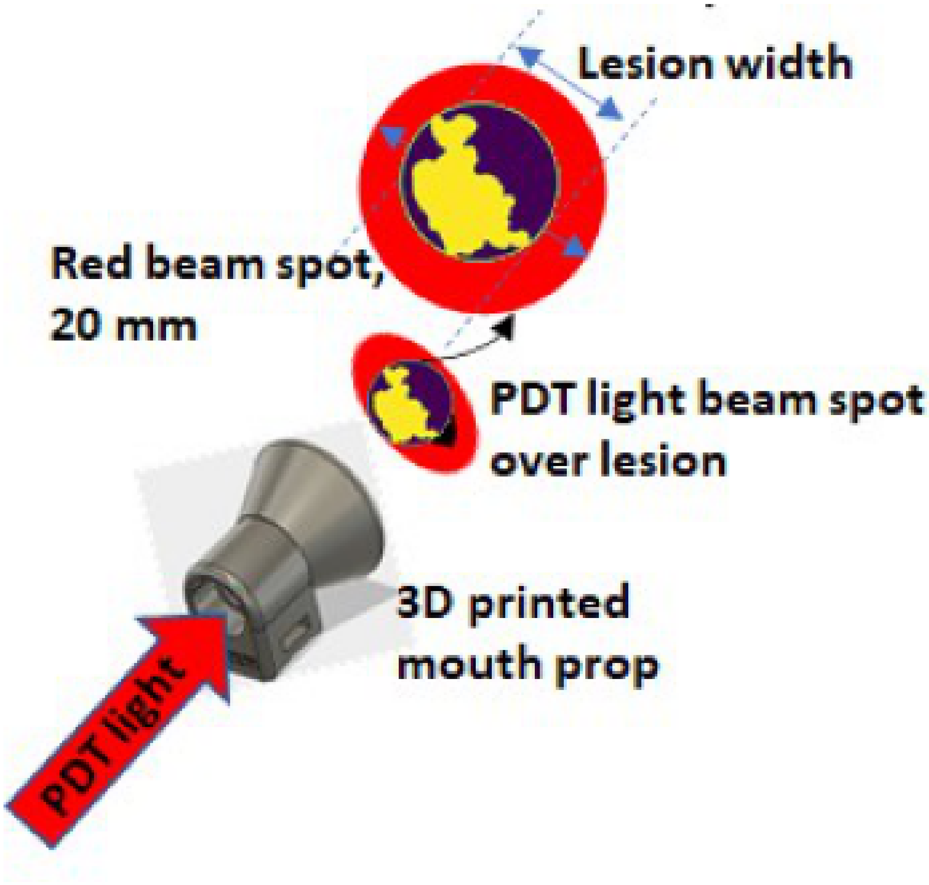

0.12 Quantification of R-value and green (auto-fluor) and red fluorescence (PpIX)

[60]:
```
im = np.array((ratio * 255)/4, dtype = np.uint8) # .............
#imshow(im)
ret, thresh1 = cv2.threshold(im, 89, 255, cv2.THRESH_BINARY)
```

```
#plt.imshow(thresh1)

thresh2=thresh1.copy()

kernel = cv2.getStructuringElement(cv2.MORPH_ELLIPSE, (30,30))
opening = cv2.morphologyEx(thresh2, cv2.MORPH_OPEN, kernel, iterations=2)

contours, hierarchy = cv2.findContours(opening,cv2.RETR_TREE, cv2.
 ↪CHAIN_APPROX_NONE)

mask = np.zeros(real_img.shape, dtype=np.uint8)

if (i, areaContour) == max((i,cv2.contourArea(c)) for i,c in␣
 ↪enumerate(contours)):
    cv2.drawContours(mask,contours,i,(255,255,255),thickness=cv2.FILLED)

    (x,y),  radius = cv2.minEnclosingCircle(contours[i])
    #cv2.circle(mask, (int(x), int(y)), int(radius), (255,225,0), 10)
    pix_per_mm=int(54.6)
    maximum_width=2*((radius/int(pix_per_mm)))
    r_10=int(pix_per_mm*10)

    R_=r_10-radius
    new_radius=R_+ radius
    #cv2.circle(mask, (int(x), int(y)), int(new_radius), (255,0,0), 40)

    beam_maximum_width=2*((new_radius/int(pix_per_mm)))

    mask=cv2.flip(mask, 1)
    mask = cv2.rotate(mask, cv2.ROTATE_180)

    #plt.imshow(mask)
    #plt.title('at thresh. r value 1.4')

    real_img = cv2.imread(real_image)
    real_img = cv2.cvtColor(real_img, cv2.COLOR_BGR2RGB)
    real_img=cv2.flip(real_img, 1)

    real_img = cv2.rotate(real_img, cv2.ROTATE_180)
#     plt.imshow(real_img)
#print(real_img.shape)
    im_thresh_color = cv2.bitwise_and(real_img, mask)
#     plt.imshow(im_thresh_color)
    R_mask, G_mask, B_mask = cv2.split(im_thresh_color)

    ratio_mask=R_mask/G_mask
#plt.imshow(ratio_mask)
```

```
    crop_above=ratio_mask[ratio_mask> 1.4]
    print("R-value under the mask",crop_above[~np.isinf(crop_above)].mean())

    R_real, G_real, B_real = cv2.split(im_thresh_color)


    crop_above=R_real[R_real> 0]
    red_int=crop_above[~np.isinf(crop_above)].mean()



    crop_above=G_real[G_real> 0]
    green_int=crop_above[~np.isinf(crop_above)].mean()

    print(f'red intensity: {red_int} and green intensity; {green_int}')

# plt.imshow(G_real)

    fig = plt.figure(figsize=(16,16))
    ax0 = fig.add_subplot(1,3,1)
    ax0.imshow(im_thresh_color)
    ax0.set_title("real masked image")
    ax1 = fig.add_subplot(1,3,2)
    ax1.imshow(R_real)
    ax1.set_title("Red channel")
    ax3 = fig.add_subplot(1,3,3)
    ax3.imshow(G_real)
    ax3.set_title("Green channel")
```

```
R-value under the mask 2.0891794096728615
red intensity: 94.20877676595187 and green intensity; 45.639885365708984
```

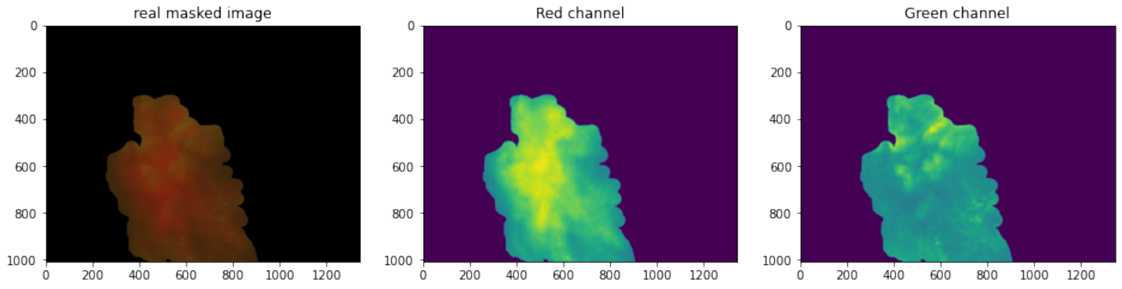

Supplement: Supplementary file 1 [file JBO_028_082809_SD001.pdf]
